# Siderophore Biosynthesis but Not Reductive Iron Assimilation Is Essential for the Dimorphic Fungus *Nomuraea rileyi* Conidiation, Dimorphism Transition, Resistance to Oxidative Stress, Pigmented Microsclerotium Formation, and Virulence

**DOI:** 10.3389/fmicb.2016.00931

**Published:** 2016-06-16

**Authors:** Yan Li, Zhongkang Wang, Xuee Liu, Zhangyong Song, Ren Li, Changwen Shao, Youping Yin

**Affiliations:** Chongqing Engineering Research Center for Fungal Insecticides, School of Life Science, Chongqing UniversityChongqing, China

**Keywords:** siderophore, a high-affinity iron permease, microsclerotia, *Nomuraea rileyi*, ROS detoxification

## Abstract

Iron is an indispensable factor for the dimorphic insect pathogenic *Nomuraea rileyi* to form persistent microsclerotia which can replace conidia or blastospores for commercial mass production. There are two high affinity iron acquisition pathways in *N. rileyi*, siderophore-assisted iron mobilization and reductive iron assimilation systems. Transcription of the two iron uptake pathways related genes is induced under iron-limiting conditions. Stage-specific iron uptake-related genes expression during microsclerotia development shows siderophore-mediated iron acquisition genes are rigorously upregulated specifically during the formation and mature period while reductive iron assimilation related genes just display a higher expression at the late maturation period. Abrogation of reductive iron assimilation, by the deletion of the high affinity iron permease (*NrFtrA*), has no visible effect on microsclerotia biogenesis in *N. rileyi*. In sharp contrast, *N. rileyi* L-ornithine-N^5^-monooxygenase (*NrSidA*), required for synthesis of all siderophores, is absolutely necessary for the development of pigmented microsclerotia. In agreement with the lower intracellular iron contents of microsclerotia in Δ*NrSidA* strains, not only the pigments, but both the number and the biomass are also noticeably reduced. Certain concentration of ROS is required for promoting microsclerotia biogenesis. Combined with expression pattern analysis of related genes and quantitative of intracellular iron or extracellular siderophore in WT and mutants, these data demonstrate the lack of adequate intracellular iron caused by the loss of the siderophore results in the deficiency of ROS detoxication. Furthermore, Δ*NrSidA* strains show significantly increased sensitivity to hydrogen peroxide. Besides, *NrSidA*, but not *NrFtrA*, play a crucial role in vegetative growth under iron-limiting conditions, conidiation, and dimorphic switching. Remarkably, the slower growth of the Δ*NrSidA* strains *in vivo* due to a reduced capacity for iron acquisition leads to the loss of virulence in *Spodoptera litura* while the Δ*NrFtrA* mutants behaved as WT during infection. Together, these results prove siderophore-assisted iron mobilization is the major pathway of cellular iron uptake and essential for conidiation, dimorphism transition, oxidative stress resistance, pigmented microsclerotium formation and full virulence.

## Introduction

To control the damage of pests to agriculture, widespread and continuous use of chemical insecticides causes environmental problems and leads to the development of insect resistance. Microbial insecticides such as entomopathogenic fungi can provide an alternative, more environmentally friendly option. Unlike insect-pathogenic viruses, bacteria, and protozoa, which must be ingested to infect a host, most entomopathogenic fungi infect by penetrating the host cuticle (Srisukchayakul et al., 2005). As a dimorphic insect pathogenic fungus, *Nomuraea rileyi* can infect many key crop pests in nature worldwide, especially noctuids such as *Helicoverpa armigera, Spodoptera litura, Tricoplusia ni, Anticarsia gammatalis*, and *Pseudoplusia* and has a potential for development into mycoinsecticide (Shanthakumar et al., 2010; Thakre et al., 2011). However, lack of reliable and cost effective substrate limits the mass cultivation and commercialization of these mycoinsecticides. Maltose and stimulatory light are required for *N. rileyi* sporulation, which results in high costs that limits the large-scale production of *N. rileyi*. Besides, the short shelf life of the most conidiospores prevent it from becoming a commercial product. Hence, an alternative method should be sought for commercial production of *N. rileyi*.

*N. rileyi* has been reported to successfully produce environmentally persistent microsclerotia (MS) (Yin et al., 2012). These MS propagules, with a diameter of 50–600 μm, are compact aggregations of hyphae that often become melanized as they develop, and produced under specific submerged liquid culture conditions (Song et al., 2013). These MS were desiccation tolerant with excellent storage stability following air-drying. Upon hydration, MS are capable of producing many infective conidia sporogenically (Jackson and Jaronski, 2009; Jackson et al., 2010; Song et al., 2015). Fungal pigments are able to act as anti-desiccants, enhance cell rigidity and protect fungi against different exogenous stresses, such as UV-irradiation, elevated temperatures, reactive oxygen species, and also against fungicide, all properties that would enhance the vigor of MS propagules for use as a mycoinsecticide in the rhizosphere (Zhong et al., 2008; Jackson and Jaronski, 2009; Heinekamp et al., 2013). Other entomopathogenic fungi such as *Cordyceps, Hirsutella, Metarhizium* species and *Lecanicillium lecanii* have also been reported to produce MS (Jackson and Jaronski, 2009; Behle et al., 2013; Wang et al., 2013). MS granules have been tested for the control of pests such as the sugar beet root maggot, *Tetanops myopaeformis*, Asian Longhorned Beetles (Coleoptera: Cerambycidae) and black-legged ticks, *Ixodes scapularis* Say (Jaronski and Jackson, 2008; Jackson and Jaronski, 2009; Behle et al., 2013; Goble et al., 2015). Although the ability to produce dry granules instead of conidia or blastospores represents an innovation for commercial interests, the mechanisms underlying the key developmental events of entomopathogenic fungi remain unresolved.

MS development is controlled by a network of signaling pathways, which must occur in concert with other complex signaling events (Song et al., 2013). To study the mechanisms of MS development, comparative transcriptome analysis of MS development in *N. rileyi* had been performed, showing that oxidative stress occurs during the physiological process (Song et al., 2013). Multiple genes of *N. rileyi* have been identified as involved in MS development (Song et al., 2013; Jiang et al., 2014; Liu et al., 2014; Zhou et al., 2015). *RacA* and *Cdc42*, small GTPases, are required for MS formation through the control of ROS generation (Jiang et al., 2014). Both NADH: flavin oxidoreductase/NADH oxidase gene (*Nox*) and alternative oxidase gene were proved to play a role in MS differentiation by regulating intracellular H_2_O_2_ concentration (Liu et al., 2014; Zhou et al., 2015). Silencing mutants of two transmembrane proteins, *Sho1p* and *Sln1p*, resulted in severely reduced MS yields and conidiation production (Song et al., 2015). In addition, iron was found to be an indispensable for the MS formation. Furthermore, the yield of the MS increased with the rising iron concentrations of the fermentation broth within a certain range (Song et al., 2014). On the basis of previous results, further studies should be focused on the roles of the iron and related genes play in the MS formation.

As an essential nutrient, iron is required for various metabolic processes, including electron transport and redox reactions in nearly all organisms (Haas et al., 2008). Fungi have evolved various strategies, often used in parallel, to acquire iron. More detailed information on the iron uptake mechanisms is available in the model fungus, *Aspergillus fumigatus. A. fumigatus* possesses two high affinity iron uptake mechanisms: reductive iron assimilation (RIA) and siderophore-mediated iron uptake (Schrettl et al., 2004). RIA involves reduction of ferric to ferrous iron and subsequent uptake of ferrous iron by the FtrA/FetC complex, an activity that is blockable with the ferrous iron-specific chelator bathophenanthroline disulfonate (BPS) (Haas, 2003; Schrettl et al., 2007). Siderophores are low molecular mass, ferric iron-specific chelators, which are excreted during iron starvation (Haas, 2003). The majority of fungal produce hydroxamate-class siderophores (Haas, 2014) but are able to take up other types of siderophores as well, such as *Saccharomyces cerevisiae* (Philpott et al., 2002). *A. fumigatus* produces four hydroxamate siderophores all based on L-ornithine-N^5^-monooxygenase (SidA) (Moore, 2013), which catalyzes both intra- and extracellular siderophore biosynthesis at initial biosynthetic step (Schrettl et al., 2007). And then the formation of the hydroxamate group is accomplished by N^5^-acylation of N^5^-hydroxyornithine with the N^5^-transacylases (SidF) (Schrettl et al., 2007; Haas et al., 2008). SidF homologs can be found among hydroxamate- producing fungi and numerous bacterial species, for example, the *Escherichia coli* homolog IucB for synthesis of siderophore aerobactin (de Lorenzo et al., 1986; Schrettl et al., 2007; Haas et al., 2008) and the potential siderophore-biosynthetic transacylase Fer5 from *Ustilago maydis* (Eichhorn et al., 2006). After the addition of an acyl moiety to N^5^-hydroxyornithine, the hydroxamates are covalently linked via ester or peptide bonds to form the final siderophore accomplished by nonribosomal peptide synthetases (NRPSs) (Haas et al., 2008). Different NRPSs appear to be responsible for producing different siderophores. In *A. fumigatus, SidC* or *SidD* takes part in the biosynthesis of intracellular siderophores FC or extracellular siderophores TAFC, respectively (Schrettl et al., 2007). Siderophore-iron chelates are usually taken up through transporters of the UMF/SIT subfamily of the major facilitator superfamily (Haas et al., 2008). In *S. cerevisiae*, the transporter Sit1p/Arn3p recognizes the bacterial hydroxamate ferrioxamine B, coprogen, and a variety of ferrichromes. *S. pombe* Str2 or Str1 has been suggested to transport ferrichrome and ferrioxamin B or ferrichrome, respectively (Ardon et al., 2001; Pelletier et al., 2002).

The function of siderophore-mediated iron acquisition and RIA in entomopatho- genic fungi is not well understood. In *N. rileyi*, iron stimulates the formation of MS at some range of concentrations and transcriptome analysis revealed that most of the genes involved in iron uptake were found to be upregulated during MS development (Song et al., 2013, 2014). Hence, a deeper understanding of the role of iron and its uptake related genes play in the corresponding physiological process will facilitate to guide the liquid fermentation of MS in *N. rileyi*. In present study, we mainly analyzed the roles of two essentially important genes (*NrSidA* and *NrFtrA*) played in MS formation, spore production, dimorphism transition and the virulence against *Spodoptera litura* and the influence on the transcriptional regulation of several genes implicated in MS formation.

## Materials and methods

### Strains, media, and culture conditions

*N. rileyi* CQNr01 was isolated from cadavers of *Spodoptera litura* infected naturally and stored at the Engineering Research Center for Fungal Insecticides, Chongqing University, China. The fungal strain and engineered strains generated in this study were grown on Sabouraud maltose agar with 1% yeast extract (SMAY) under continuous illumination at 25°C for 14 days to produce conidia. The conidia suspensions were prepared in sterile 0.5% Tween 80 (Sigma-Aldrich, USA). The spores of *N. rileyi* was incubated in liquid-amended medium (AM) (containing 40 g/L of glucose, 2.5 g/L of peptone, 5 g/L of yeast extract, 4.0 g/L of KH_2_PO_4_, 0.8 g/L of CaCl_2_.2H_2_O, 0.6 g/L of MgSO_4_.7H_2_O, 0.1 g/L of FeSO_4_.7H_2_O, 37 mg/L of CoCl_2_.6H_2_O, 16 mg/L of MnSO_4_.H_2_O, and 14 mg/L of ZnSO_4._7H_2_O) at 28°C under shaking at 250 rpm for MS production. Phenotypic characterizations were carried out using strains grown on *Aspergillus* minimal medium (AMM) amended with 4% (wt/vol) maltose as carbon source, 20 mM glutamine as nitrogen source, and 10 μM FeSO_4_.7H_2_O or FeCl_3_.7H_2_O when required. For iron-depleted conditions, iron was omitted.

### Gene cloning and bioinformatic analyses

To study the potential functions of *NrSidA* and *NrFtrA* on the MS development, the full cDNA sequences were acquired using fusion primer and nested integrated PCR (FPNI-PCR) and RACE based on the ESTs from the transcriptome (Liu and Chen, 2007; Wang et al., 2011; Song et al., 2013). Both deduced amino acid sequences were used for Blastp searches against GenBank. The homologs of the two genes from other fungal species were collected from the NCBI database for multiple sequence alignment analysis separately with ClustalX (Larkin et al., 2007) and a neighbor- joining tree was generated with 1000 bootstrap replicates using the program MEGA 7.0 (Tamura et al., 2013).

### Generation of *NrSidA* and *NrFtrA* mutants

For inactivation of *NrSidA* and *NrFtrA* through homologous recombination, respective flanking regions were obtained from genomic DNA by FPNI-PCR (Wang et al., 2011) with the primers listed in Table S1 and inserted in to pMD19-T vector for sequencing.

To generate the *NrSidA* and *NrFtrA* disruption vectors, upstream and downstream flanking sequences were obtained by PCR with primers listed in Table S1. The resulting upstream flanking sequence of the *NrSidA* or *NrFtrA* was digested with *Eco*RI/*Xho*I or *Bam*HI/*Xba*I and with *Xba*I/*Hind*III or *Pst*I for the downstream flanking sequence and then inserted into pPZP-Hph vector harboring a hph cassette to yield pPZP-Hph-NrSidA or pPZP-Hph-NrFtrA, respectively. The resulting clones were introduced into *Agrobacterium* strain AGL1 for fungal transformation. *Agrobacterium tumefaciens* mediated transformation (ATMT) of *N.rileyi* CQNr01 with pPZP-Hph-NrSidA or pPZP-Hph-NrFtrA was performed as described elsewhere (Shao et al., 2015).

Transformants resistant to the hygromycin were selected. Initial screening of transformants for the disruption of the *NrSidA* or *NrFtrA* gene was carried out by PCR with the primers listed in Table S1. Gene deletion was further assessed in single conidial progenies by Southern analysis according to the instruction of the DIG High Prime DNA Labeling and Detection Starter Kit I (Roche, Mannheim, Germany). All primers used in construction and verification of engineered strains are listed in Table S1.

### Quantification of intracellular iron in MS and LIP determination in yeast-spores

Intracellular iron concentration in MS was measured using the BPS-based colorimetric assay (Epsztejn et al., 1997; Tamarit et al., 2006) with modifications. Briefly, 3.5-day-old MS was harvested by filtration, washed three times with ddH_2_O, ground with liquid nitrogen, weighed after vacuum freeze drying and resuspended in 1000 μL of 3% nitric acid. Suspensions were boiled for 8 h and centrifuged at 12,000 g for 15 min to discard cell debris. A total of 100 μl of supernatant was diluted 10-folds (except the MS cultivated in AM-Fe medium) and then 400 μL of supernatant diluent was mixed with 160 μL of sodium ascorbate, 320 μL of BPS, and 126 μL of ammonium acetate, and reactions were incubated at room temperature for 5 min. OD_535_ of the BPS-Fe complex was measured with the DU®640 Speterophozometer (Beckman coultor, USA). To eliminate the nonspecific absorbance, OD_680_ was subtracted from OD_535_. Iron was quantified taking into account a calibration curve using seven solutions with known FeSO_4_ concentrations (0.02–0.16 mM; y = 0.127x-0.003; *R*^2^ = 0.996), and the results were expressed as μmol g^−1^dry weight. Three replicates were performed, and each replicate was measured in triplicate.

The labile iron pool (LIP) in yeast-spores is measured by using calcein, a fluorescent probe that binds to both Fe^2+^ and Fe^3+^ (Epsztejn et al., 1997). The binding of Fe quenches the fluorescence of calcein. After cellular uptake of calcein-AM, intracellular esterases convert the molecule into a non-permeable acid-form of calcein (Cabiscol et al., 2002). *N. rileyi* strains yeast-spores were obtained after growing conidiophores on the SMAY for 4 days. Yeast-spores were washed twice with phosphate-buffered saline (PBS), incubated for 3 h at 37°C in 5 μM Calcein-AM (a final concentration) in PBS, and then washed twice for 5 min with PBS to remove unincorporated dye. Fluorescence was observed (excitation and emission at 485 nm and 530 nm, respectively) under a Nikon fluorescence microscope (Nikon, Japan).

### Chrome azurol S (CAS) liquid assay and quantification of siderophores

Extracellular siderophores was determined using the chrome azurol S liquid assay as described (Schwyn and Neilands, 1987; Payne, 1994; Louden et al., 2011). One hundred microliter of the CAS solution (10 ×) was mixed with 900 μl the culture filtrates from the 3.5 day-old MS growing in AM. Siderophore production was further measured by a Csaky test (Csaky, 1948). 0.5 mL of culture filtrates were mixed with 2.5 mL of iron (III) perchlorate (5 mM) dissolved in 0.1 M perchloric acid and measured spectrophotometrically for absorbance at 480 nm.

### Purification and measurement of MS pigments

The MS melanin/pigment was isolated from 3.5-day-old cultures with 2%NaOH. The collected MS was ground with liquid nitrogen, suspended in NaOH solution (1 mL) and boiled at 100°C for 2 h. The solution was acidified to pH 2.0 with 5 M HCl and centrifuged at 6000 g for 15 min. The resulting melanin/pigment was dissolved in 2% NaOH, and the solution was measured spectrophotometrically for absorbance at 459 nm (Babitskaya et al., 2000).

### Phenotypic analyses

The sensitivity to NaCl, sorbitol, sodium dodecyl sulfate (SDS), calcofluor white (CFW), congo red (CR) and various high concentration metal ions including CaCl_2_, MnSO_4_, ZnSO_4_, and CuSO_4_ was tested in AMM plus 1.8% agarose as the solidifying agent. Conidial suspensions (5 × 10^5^ conidia) were pipetted onto plates and incubated at 25°C and inspected regularly for 14 days for colony morphology and diameter. Heat tolerance was determined according to Liu et al. (2010).

The sensitivity of conidia to killing by hydrogen peroxide was assayed under different consideration iron. As mentioned above, 5 × 10^5^conidia of WT and mutants were point inoculated and radial growth was measured after 14 days at 25°C on AMM medium without iron (−Fe) or with 10 μM FeSO_4_ (Fe) or 1.5 mM FeSO_4_ (hFe), respectively, and with the addition of 0.25 mM bathophenanthroline disulfonate (BPS) or/and 2 mM H_2_O_2_. The AMM medium without H_2_O_2_ was as a control.

The sensitivity of conidia to hydrogen peroxide with different concentrations was assayed as described by Schrettl et al. (2007). Briefly, conidial suspensions approximating 10^5^ conidia ml^−−1^ were incubated for 30 min at room temperature with varying concentrations of hydrogen peroxide. To determine the number of surviving conidia, the spore suspensions were diluted 50-fold and plated on SMAY. Following incubation for 6 days at 25°C, colonies were counted and normalized to that without hydrogen peroxide treatment. The sensitivity of hyphae to hydrogen peroxide was estimated by using a modification of the protocol of Kawasaki et al. (1997). Approximately 100 conidia were plated on SMAY medium and grown at 25°C for about 8 day for the countable colonies formation and at this time each colony grew out the fungal mycelia. Subsequently, the plates were overlaid with 8 ml of the same medium as top-agar but containing the indicated concentration of hydrogen peroxide. After further incubation for 5 days at 25°C, colonies able to resume growth were counted as survivors and normalized to the number before hydrogen peroxide treatment.

Sporulation rates were tested using 5 × 10^5^ conidia on AMM amended with 0, 10 μM and 1.5 mM FeCl_3_ or FeSO_4_, respectively. The plates were incubated at 25°C for 14 days for sporulation and the numbers were counted. Besides, the sporulation rates were also recorded at intervals of 3–11 days using an equal number conidia on SMAY.

Germination tests were conducted using 1 × 10^5^ conidia on SMAY or AMM amended with (250 μM) BPS, 0 or 30 μM FeSO_4_. Cultures were incubated at 25°C and the conidial germination rates at various time-points were determined by counting 150 conidia. The experiment was performed three times; each involved three replicates.

*N. rileyi* displays a dimorphic switching, using the yeast cell form to avoid phagocytosis and the cytotoxic environment of the phagolysosomal system (Wanchoo et al., 2009; Boyce and Andrianopoulos, 2015). *In vitro, N. rileyi* can grow in yeast-cell form at the early stage (3~6days) on SMAY and then gradually transform into filamentous form. Switching rates from yeast-cell to hyphal were studied using 1 × 10^5^ yeast-cells on SMAY and regularly observed for colony morphology. In addition, approximately 80 simple yeast-cells were plated on SMAY medium, grown at 25°C and the switching rates at various time-points were recorded. The median transition rates time (TT_50_) of the mutants were compared with WT.

### Insect virulence assays

Virulence of the WT, Δ*NrSidA* and Δ*NrFtrA* was assayed against newly emerged second-instar *Spodoptera litura* larvae. Conidia from SMAY plates were applied topically on the abdominal dorsum in a mineral oil suspension containing 1 × 10^8^ conidia ml^−1^. Each treatment had three replicates with 15 insects each, controls were treated with mineral oil only, and the experiments were repeated three times. For injection assays, each insect was injected from the second proleg with 10 μl of an aqueous suspension containing 1 × 10 ^7^ conidia ml^−1^. The number of dead insects was recorded daily for 10 days. The median lethal time (LT_50_) was calculated using a probit analysis with the SPSS program.

Hyphal bodies represent a yeast-like fungal cell type produced in the insect hemolymph after hyphal penetration through the integument to evade the insect immune system (Wanchoo et al., 2009). For collection of hemolymph, infected and control larvae were anesthetized on ice and the rear leg was cut off with a scissor at 72, 96, and 132 h after injected with 10 μl of an aqueous suspension containing 2.5 × 10^7^conidia ml^−1^. The exuded hemolymph from the wound was collected. Fungal hyphal bodies present in the insect hemolymph were observed via bright-field microscopy (Olympus, Japan), and the number of hyphal bodies was determined by direct counting using a hemocytometer. Three replicates were performed for each treatment, and each replicate contained three randomly picked larvae.

### Gene expression by quantitative reverse transcription polymerase chain reaction (qRT-PCR)

In order to induce iron starvation, conidia from the WT were grown in liquid AMM lacking iron (−Fe) for 2 days, and then transferred to fresh AMM containing 0, 0.03 or 1.5 mM FeSO4, or 0.25 mM BPS for an additional day. Subsequently, the mycelia were harvested for RNA extraction. For time-specific expression patterns during MS development, 10^9^ conidia of the WT, Δ*NrSidA* and Δ*NrFtrA* were inoculated in AM and the cultures were collected by filtration and washed three times with sterile distilled water at 36, 48, 60, 72, 84, 98, 120, and 144 h for RNA extraction. To study the effects of the different consideration irons on growth and formation of MS, 10^9^ conidia of the WT, Δ*NrSidA* and Δ*NrFtrA* were inoculated in AM for 3.5 days under different iron concentrations and collected for RNA extraction. To explore the impacts of the hydrogen peroxide on the pathways of the iron uptake in MS, 3.5-day-old MS was treated with varying concentrations of hydrogen peroxide for 30 min and then collected for transcriptional analysis.

Total RNA from *N. rileyi* was isolated using Trizol reagent (TAKARA, Japan) and then treated with DNase I (Thermo Fisher Scientific, USA). cDNA of each sample was generated using RevertAid First Strand cDNA Synthesis Kit (Thermo Fisher Scientific, USA). Amplification mixtures (20 μl) for qRT-PCR contained 2 μl template cDNA, 10 μl 2 × IQ SYBR Green Supermix (Bio-Rad, USA) and 1 μl of each primer (PF/PR, 10 μmol l^−1^). The reaction profile was performed as an initial 95°C for 3 min, followed by 42 cycles of 95°C for 10 s, appropriate annealing temperature for 20 s and 72°C for 20 s. PCR reaction was performed using the iCycler system (CFX, Bio-Rad) and primers listed in Supporting Information Table S1. qRT-PCR efficiency was determined by 10-fold gradient dilutions of cDNA of each target sequence for standard curve production. Under the optimal annealing temperature, the calculated efficiency of all primers was 95–105%. Melt curve analyses were carried out to confirm the absence of the nonspecific products. Relative expression of each gene was normalized against the expression of a β-tubulin gene (TUB) and a transcription elongation factor (TEF). The resulting data were analyzed with the CFX qPCR software. All PCR amplifications were conducted in triplicate, and trials were repeated three times.

### Data analysis

Unless otherwise indicated, all data were analyzed by one-way ANOVA, followed by Duncan's Multiple Range test using the SPSS 17 program (SPSS 17.0, SPSS Inc, USA). Uppercase or lowercase letters indicated the statistically significant level at *P* < 0.01 or *P* < 0.05, respectively. Means indicated by the same letter are not significantly different from one another.

## Results

### Molecular characterization of *NrSidA* and *NrFtrA*

Two fragments that showed strong sequence similarity to fungal genes encoding L-ornithine-N^5^-monooxygenase (SidA) or a high-affinity iron permease (FtrA) separately were identified from the expression profiles due to the up-regulated expression in the transcriptome of MS. Both entire cDNA sequences were amplified by FPNI-PCR and RACE and designated as *NrSidA* (GenBank accession number:KX181541) or *NrFtrA* (accession number:KX181542), respectively. The putative coding sequences of *NrSidA* and *NrFtrA* were 1863 and 1080 bp, encoding 620 and 359 amino acid residues with a calculated molecular weight of 66.97 and 38.84 kDa and an isoelectric point of 5.85 and 8.89, respectively (http://expasy.org/tools/protparam.html).

The comparison of cDNA and genomic sequences revealed the presence of two introns in both genes. Both *NrSidA* and *NrFtrA* expressions are under the SreA-mediated regulation (Oberegger et al., 2001) as the promoter regions contained several HGATAR motifs (data not shown), which represent putative binding sites for GATA-factors such as SreA.

The amino acid sequence of *N.rileyi* NrsidA showed very high identity to sidA from *M. anisopliae* (81%) and *B. bassiana* ARSEF 2860 (65%) and also displayed 50 and 48% identity to SidA from *A.fumigatus* and *A.nidulans*, respectively (Figure S1A). The *N.rileyi* NrSidA sequence contained the three signature sequences typical of amino acid hydroxylase enzymes. The first of these is the flavin adenine dinucleotide binding (FAD) motif GXGXXG (^159^CVGFGP^164^) and the last glycine in this motif of NrSidA was exchanged for proline, which is a typical feature of siderophore biosynthetic enzymes (Stehr et al., 1998). Another typical feature for siderophore biosynthetic genes was a putative nicotinamide adenine dinucleotide phosphate (NADP)-binding motif GXGXX(G/A) (^365^**G**A**G**QS**A**A^371^). Besides, the conserved motif D(X)_3_(L/F)ATGY(X)_4_(H/P) (^512^**D**LVIA**ATG**YQRNA**H**^525^) was proposed to be involved in substrate binding (Eisendle et al., 2003). Phylogenic analysis indicated that NrSidA protein had the closest relationship with that from the entomopathogenic fungus *Metarhizium* spp. (Figure S1D).

NrFtrA showed typical features of a high affinity iron permease protein. NrFtrA protein, the same as the *S. cerevisiae* iron permease Ftr1, contained seven transmembrane domains (Figure S1C; Kwok et al., 2006) and two motifs of REXXE, ^16^RETLE^20^ and ^158^REGIE^162^. The glutamic acid residue in REXXE motif, which was interact directly with iron, was conserved in the selected FTR protein sequences from other organisms (Figure S1B). With respect to amino acid similarity, NrFtrA displayed 59% and 48% identity to FtrA from *A. fumigatus* and Ftr1 from *R. oryzae*, respectively. Phylogenic analysis indicated that NrFtrA protein was closely related to that from the entomopathogenic fungus *Metarhizium* spp.while had rather distant phylogenetic relationships to *R. oryzae* (Figure S1E).

**Figure 1:**
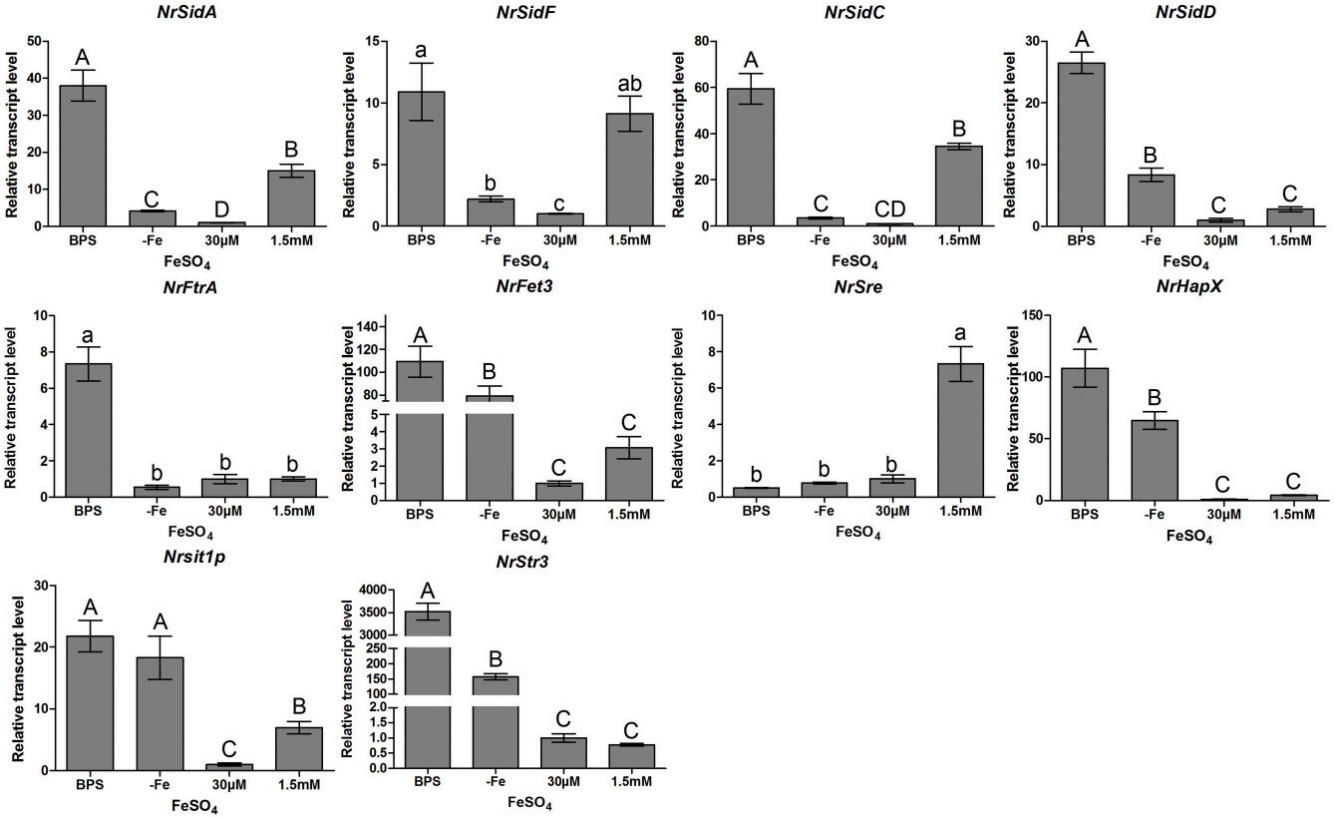
**Relative transcript abundance of iron uptake-related genes**. Relative transcript abundances of iron uptake-related genes at different iron supplies were measured by qRT-PCR. TEF and TUB transcripts were used as reference. Uppercase or lowercase letters indicated the statistically significant level at *P* < 0.01 or *P* < 0.05, respectively; the same letter were not significantly different from one another. Error bars are standard deviations of three trials.

The transcriptional response of two high affinity iron uptake pathways related genes of *N.rileyi* to iron availability was investigated by qRT-PCR in the AMM liquid medium. Under iron-limiting conditions (−Fe or BPS), *N. rileyi* wild-type strain CQNr01 induced the expression of genes involved in the biosynthesis of siderophores (*NrSidA, NrSidF, NrSidD* and *NrSidC*), uptake of siderophores (*NrSit1p* and *NrStr3*; Heymann et al., 2002; Pelletier et al., 2002) and RIA (*NrFtrA* and *NrFet3*, a multicopper oxidase; Askwith et al., 1994; Stearman et al., 1996; Kwok et al., 2006; Figure 1). High iron concentrations (30 μM or 1.5 mM FeSO_4_) efficiently down- regulated *NrSidD* and RIA genes transcripts at concentrations but led to increased transcript levels of the *NrSidA* and *NrSidC* genes required for synthesis of intracellular storage siderophores and control of intracellular iron homeostasis. As expected, under iron deletion conditions, transcripts of the GATA transcription factor NrSre, a putative repressor of genes involved in iron uptake (Chao et al., 2008; Haas, 2012), were present at low concentration while bZIP-type regulator NrHapX, required for adaption to iron starvation (Schrettl et al., 2010; Gsaller et al., 2014), showed high transcript levels (Figure 1). Increasing concentrations of FeSO_4_ to 30 μM or 1.5 mM increased transcript abundance of *NrSre* and decreased transcript concentration of *NrHapX*. *NrSre* and *NrHapX* had a strong response to iron in exactly the opposite way: iron abundance induced *NrSre* expression in wild-type *N.rileyi*, whereas iron-poor conditions induced *NrHapX* expression when grown in the AMM liquid medium (Figure 1).

### Identification of deletion mutants

Through homologous recombination and *Agrobacterium tumefaciens* mediated transformation, *NrSidA* and *NrFtrA* were individually disrupted by replacement with the hygromycin b phosphotransferase (hph) cassette (Figures S2A,B). Several mutant candidates for each of the targeted genes were identified by PCR screening. After single spore isolation, single integration of the replacement constructs were verified by PCR and Southern blot analysis (Figures S2C,D). Finally, two disruption mutants were obtained for *NrsidA* and three for *NrFtrA*. Both Δ*NrSiDA* strains displayed the same phenotype and all three Δ*NrFtrA* strains showed the same features.

### *NrSidA* is required for the biogenesis of pigment MS by regulating the siderophore production and iron uptake

**Figure 2:**
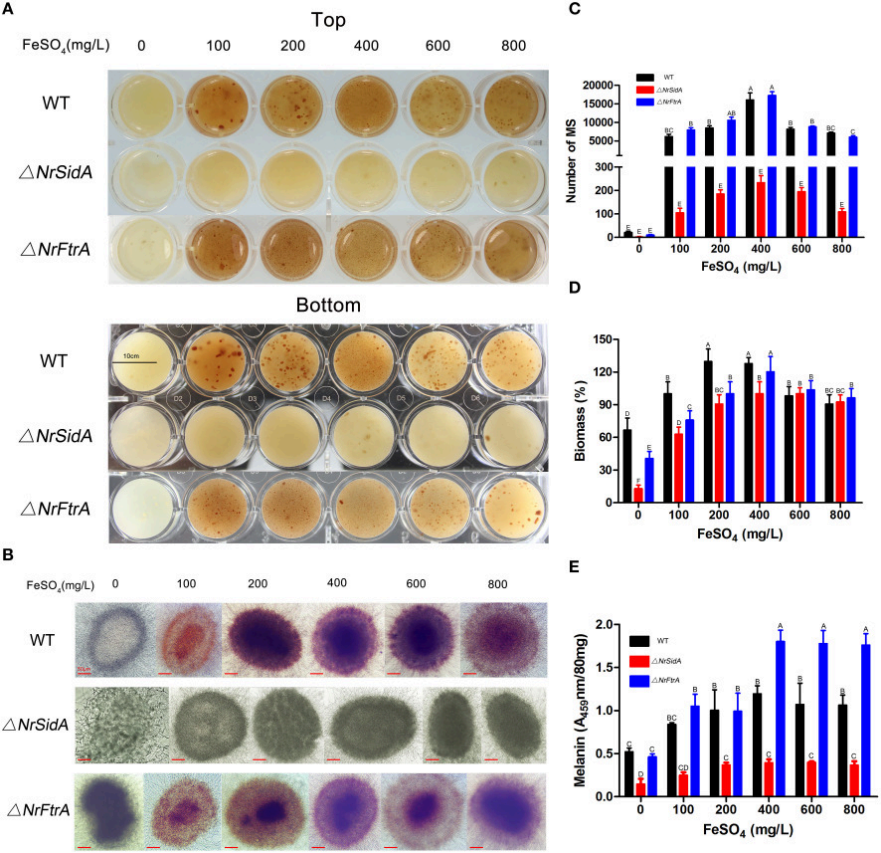
**The morphology of the MS of *N. rileyi* WT and mutants at different iron supplies and quantitative analysis of the number, biomass and melanin produced by the 3.5-days old MS in *N. rileyi* strains**. Morphology of the *N. rileyi* WT and mutants grown in AM medium at different iron supplies **(A)** or under the microscope **(B). (C)**The number of *N. rileyi* MS was counted under the optical microscope. **(D)** The equal volume of the culture of MS were washed by deionized water, dried by vacuum freezer and weighed. The biomass of the WT with 100 mg/L FeSO_4_ was as a control. **(E)** Melanin was purified with 2% NaOH and measured spectrophotometrically for absorbance at 459 nm. Each column represents the mean number of MS, relative biomass and absorbance ± standard deviation from three independent experiments, with at least three replicates. Means indicated by the same letter are not significantly different from one another, and uppercase or lowercase letters indicated the statistically significant level at *P* < 0.01 or *P* < 0.05, respectively.

To examine whether both *NrSidA* and *NrFtrA* participate in growth and MS production, conidia from the WT and mutants were inoculated in AM for MS biogenesis. After 3.5 days of induced culturing, the WT and Δ*NrFtrA* strains generated MS normally and accumulated melanin in MS, whereas the Δ*NrSidA* strains developed few MS and accumulated less melanin (Figure 2A). To ascertain whether a defect of the iron uptake caused by *NrSidA* deletion led to deficiencies in MS, conidia of the WT and mutants were inoculated in AM at different iron supplies for 3.5-days induction culture as before. Under iron deletion condition (AM-Fe), both WT and Δ*NrFtrA* mutants formed few MS and accumulated less melanin. By contrast, the Δ*NrSidA* mutants were not only pigment deficient, accumulating less melanin than WT and Δ*NrFtrA* mutants (Figures 2A,B,E) but also failed to form compact aggregations of hyphae and accumulate significant biomass (Figure 2B). With a increase in the iron concentration of AM, although the hyphae of MS in the Δ*NrSidA* strains was compact aggregations and the biomass were comparable to that of WT, the number of MS was still distinctly less than that in WT and Δ*NrFtrA* (Figures 2C,D). Furthermore, the pigments in the WT and Δ*NrFtrA* mutants could fully recover when supplemented with 100 mg/L FeSO_4_, and increased as the concentrations of iron rose. However, increasing the concentrations of FeSO_4_ to 400 or 600, even to 800 mg/L in AM just restored a portion of pigments in Δ*NrSidA* (Figures 2A,B,E), suggesting that *NrSidA* might play a vital role in the MS development especially in pigment biosynthesis.

**Figure 3:**
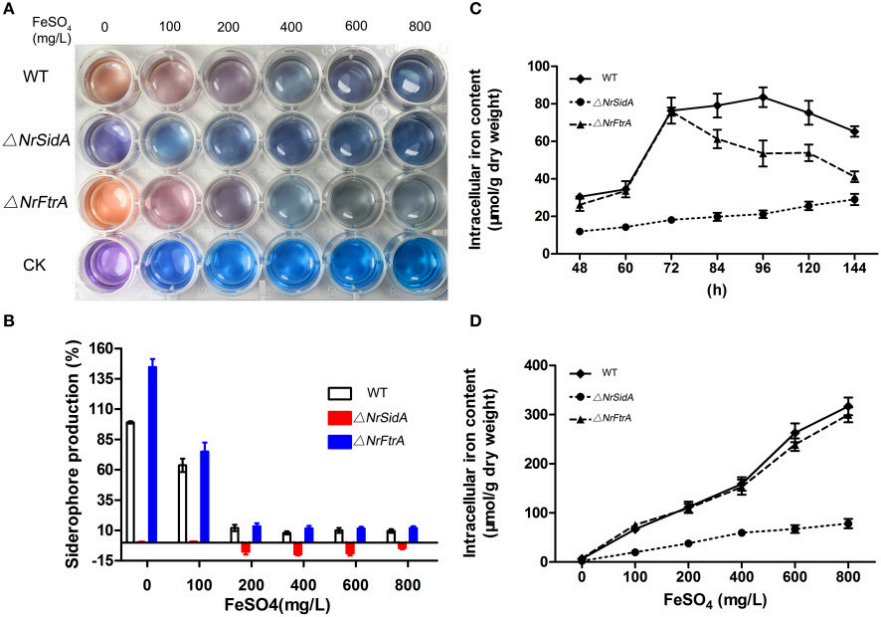
**Quantitative analysis of the extracellular siderophore and intracellular iron produced by the MS of *N. rileyi* WT and mutants. (A)** Siderophore production in *N. rileyi* WT, Δ*NrSidA* and Δ*NrFtrA* strains was measured in the culture supernatant of AM liquid culture using the chrome azurol S assay and the corresponding concentrations of FeSO_4_ were added as a control. Change from blue to pink indicates the presence of siderophores. CK: Blank **(B)** Extracellular siderophore of MS *N. rileyi* strains was quantified by a Csaky test and normalized to that of WT in AM without iron. **(C)** Quantitative analysis of intracellular iron during the MS biogenesis. **(D)** Quantitative analysis of intracellular iron of the 3.5-day-old MS under different iron concentrations.

To understand how the *NrSidA* and the *NrFtrA* genes affect the MS formation, extracellular siderophore and intracellular iron contents were measured. Under iron deletion condition, a large amount of extracellular siderophore was detected in both the Δ*NrFtrA* and WT, and then a decline appeared followed by the increase of the concentrations of iron in AM. In sharp contrast, no siderophore production was evident in the Δ*NrSidA* strains regardless of whether the iron was sufficient or deficient (Figures 3A,B). Remarkably, a much larger amount was detected in the Δ*NrFtrA* strains under iron deficient condition compared with WT (Figure 3B), indicating that Δ*NrFtrA* strains had to excreted more siderophores to remedy the missing of RIA. Meanwhile, the intracellular iron contents in different stages of the MS development and in formation period (3.5 days) with different iron concentrations were measured to evaluate the loss of siderophore on the ability of WT and mutants to acquisition and store of iron. As the MS of *N.rileyi* was developing, the intracellular iron contents in the Δ*NrFtrA*, as well as in the WT strains, gradually rose whereas the increase in the Δ*NrSidA* strains was slower (Figure 3C). Likewise, the intracellular iron contents in the Δ*NrFtrA* and WT strains rose dramatically as the iron concentrations increased whereas Δ*NrSidA* strains showed a slower increase (Figure 3D). A analogous result was found in the labile iron pool determination by the fluorescent iron dye calcein that much less free iron was tested in the yeast-spores of the Δ*NrSidA* rather than in the Δ*NrFtrA* strains (Figure S3).

### Loss of *NrSidA*, but not *NrFtrA*, leads to defects in mycelial growth and conidiation, delaying conidial germination and the dimorphic transition

**Figure 4:**
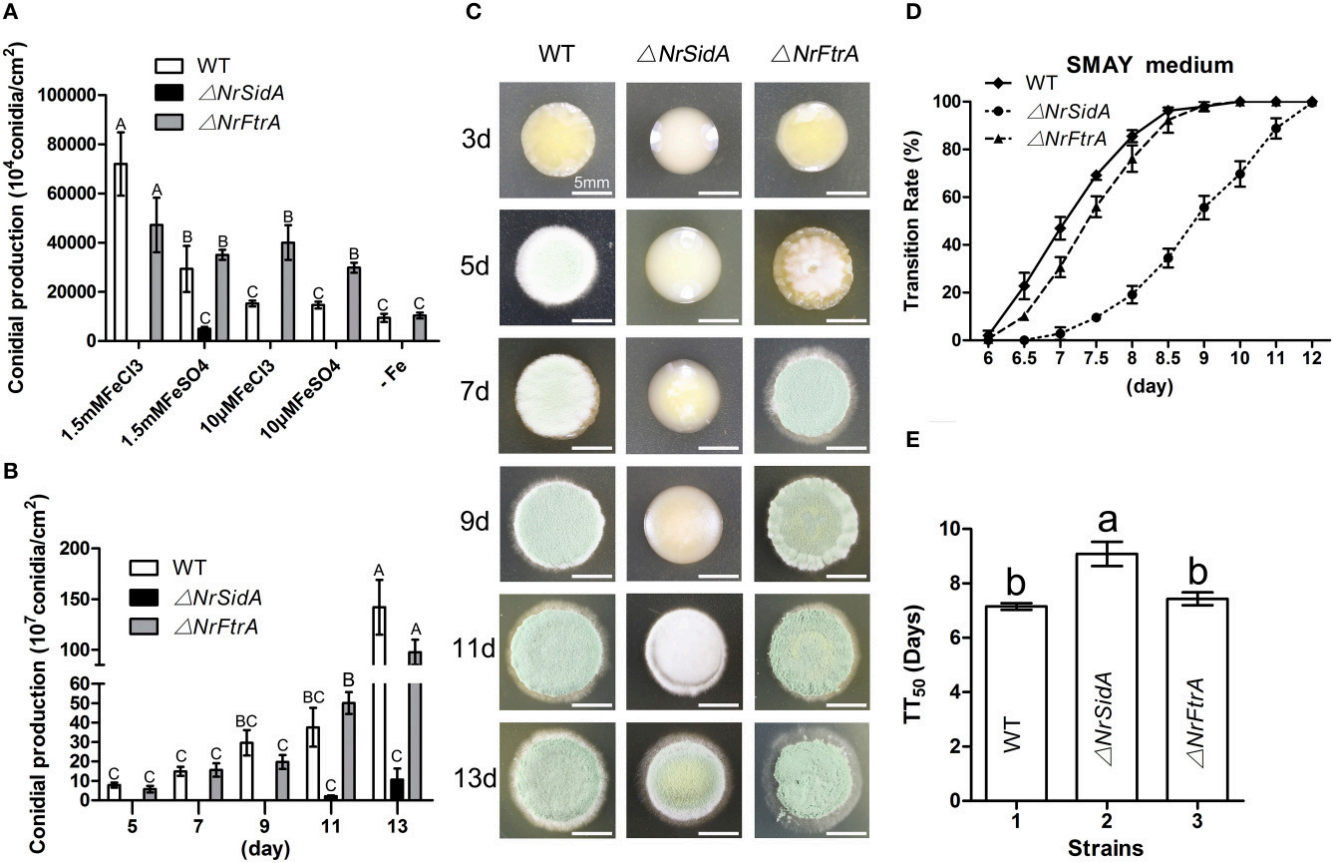
**Quantitative analysis and comparison of conidia and the dimorphic transition of *N. rileyi* WT and mutants**. Quantitative analysis of the conidia produced by *N. rileyi* WT and mutants on AMM medium at different iron supplies **(A)** or SMAY medium every 2 days **(B)**. **(C)** The dimorphic transition of the *N. rileyi* WT and mutants grown on the SMAY. Equal yeast-cells of the WT and mutants were pipetted onto plates and recorded every 2 days. Bar = 5 mm **(D)** Quantitative analysis of the dimorphic transition (from yeast-cell to hypha) rate with approximately 80 single yeast-cells plated on the SMAY medium. The growth morphology were observed every half day or a day. **(E)** The median transition rates time (TT_50_) of *N. rileyi* WT and mutants were compared. TT_50_ was calculated using a probit analysis with the SPSS program.

When grown on AMM amended with 0, 10 μM FeCl_3_ or FeSO_4_, the Δ*NrSidA* mutants were defective in sporulation while the Δ*NrFtrA* mutants behaved like WT (Figure 4A). The inability to sporulate only partially restored by the addition of 1.5 mM FeSO_4_, but not FeCl_3_. But the amount was still 100 folds less than that of WT and Δ*NrFtrA* mutants. Meanwhile, the dimorphic transition of these strains on SMAY medium was observed. The Δ*NrFtrA* mutants were comparable to that of WT: yeast-cell began to switch to mycelial and sporulated on the 5th day (Figures 4B,C). In comparison, the Δ*NrSidA* mutants showed a much slower switching : the conversion of yeast-cell to hyphae was present on the 9th day and mycelial phase started to sporulate on the 11th day, resulting in prolonging the yeast-to-mold conversion (Figures 4B,C). To eliminate the effects of the initial inoculum size on the morphologic switch, single yeast cell of the WT and mutants was grown on the SMAY medium. The similar results were showed in Figures 4D,E that the differences between WT (TT_50_ = 7.2 ± 0.1 days) and Δ*NrSidA* mutants (TT_50_ = 9.1 ± 0.4days) were significant with no obvious differences between WT and Δ*NrFtrA* mutants (*P* < 0.001), which proved once more that a delay in the dimorphic transition was indeed caused by *NrSidA* deletion.

**Figure 5:**
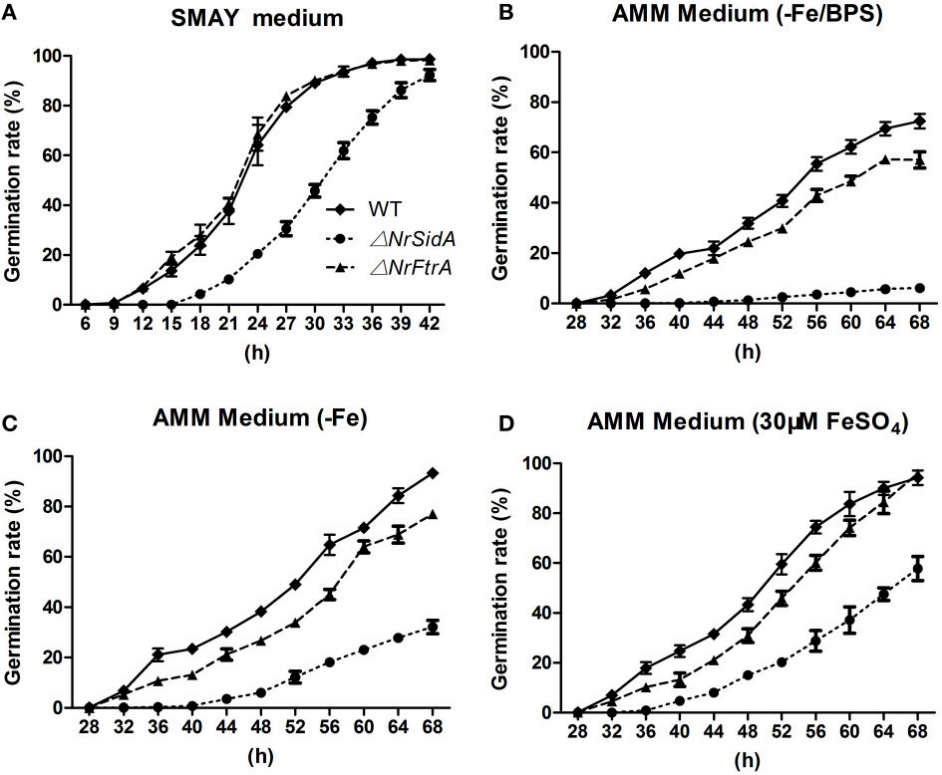
**Germination rates of *N. rileyi* WT and mutants grown on SMAY medium (A), or AMM-Fe medium (C), AMM-Fe medium supplied with BPS (B) or with 30 μM FeSO_4_(D)**.

The effects of *NrSidA* and *NrFtrA* genes disruption on conidial germination were investigated on SMAY or AMM medium. Δ*NrSidA* exhibited a significantly decreased germination either on SMAY or AMM compared with that of WT (Figure 5). When grown on SMAY medium, the germination efficiency was distinctly delayed while the rate of Δ*NrFtrA* was the same as that in WT (Figure 5A). After 24 h of incubation, about 20% of Δ*NrSidA* conidia had germinated compared with 64 or 68% of WT or Δ*NrFtrA* conidia, respectively. Similarly, after 56 h on AMM-Fe media, the germination rate was about 18% for Δ*NrSidA* conidia but 65 or 45% for WT or Δ*NrFtrA* conidia, respectively (Figure 5C); when supplemented with 30 μM FeSO_4_, the germination rates at 56 h were increased to 29% for Δ*NrSidA* conidia while 74% or 60% for WT or Δ*NrFtrA* conidia, respectively (Figure 5D). Nevertheless, the Δ*NrSidA* conidia under iron depletion in the presence of BPS nearly failed to germinate and after 56 h, the germination rates only reached 4% compared to 55 or 43% for WT or Δ*NrFtrA*, respectively (Figure 5B). These observations suggested that siderophore- mediated iron storage or utilization of intracellular iron is required for efficient growth initiation under iron limitation.

### Δ*NrSidA* mutants, but not Δ*NrFtrA* are hypersensitive to iron starvation, oxidative stress and other abiotic stresses

**Figure 6:**
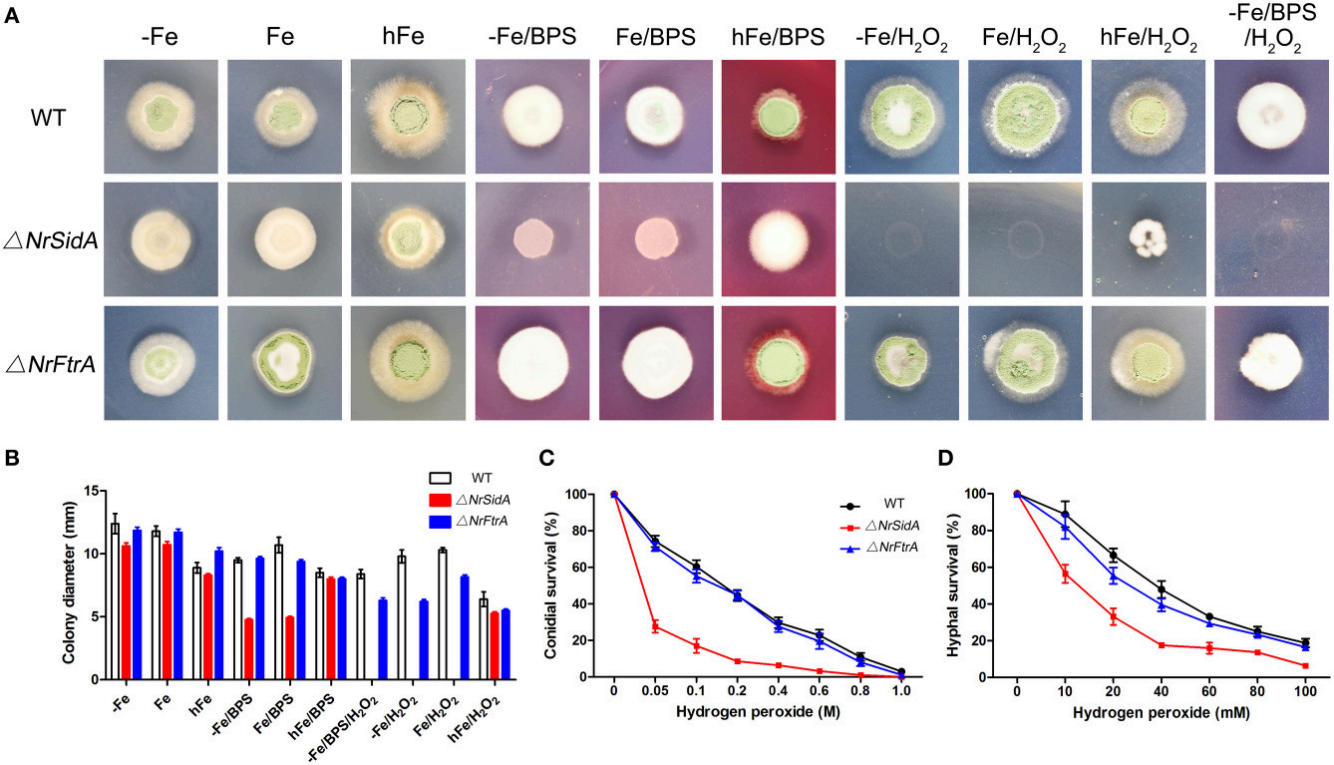
**Impact of siderophores on resistance to iron limitation and oxidative stress. (A)** 5 × 10^5^conidia of *N. rileyi* WT and mutants were point inoculated on minimal medium lacking iron (−Fe), containing 30 μM FeSO_4_(Fe), or 1.5 mMFeSO_4_(hFe), or 0.25 mM bathophenanthroline disulfonate (BPS), or 2 mM H_2_O_2_, respectively. **(B)** Colony diameter of the WT and mutants were measured after 14 days at 25°C. The data represent the means ± standard deviations of results from three independent experiments. **(C,D)** Analysis of hydrogen peroxide sensitivity of conidia **(C)** and hyphae **(D)** was determined as described in Materials and Methods. The conidia used were harvested from SMAY medium. Samples were prepared in triplicate.

The resistance of WT and mutants to iron starvation was studied under iron-sufficient and deficient conditions. In all assays performed as before, the growth rates of both genes mutants were comparable to that of WT except that the growth rate in Δ*NrSidA* was reduced significantly under iron-depleted conditions (AMM+BPS or AMM+Fe+BPS) (Figure 6A).

The effects of the other high concentration metal ions on the growth of the WT and mutants were also investigated. The susceptibility of high concentration CaSO_4_, MnSO_4_ and CuSO_4_ ions to both genes mutants as well as to WT except zinc (Figure S4B). When treated with 10 mM ZnSO_4_, Δ*NrSidA* was significantly more sensitive to zinc ion than Δ*NrFtrA*. As the concentration of ZnSO_4_ increased 2.5-fold or 5-fold, these mutants were as sensitive as WT (Figure S4B), suggesting that zinc appeared to affect iron uptake by interfering with RIA pathway.

In addition, the resistance of NaCl, sorbitol and heat to these mutants was as well as to WT when grown on normal medium and non-biotic stress but the Δ*NrSidA* mutants were slightly sensitive to the cell wall disturbing agent (SDS, CFW and CR) (Figure S4).

Inappropriate iron storage can catalyze formation of reactive oxygen species (ROS) and detoxification of hydrogen (peroxide) depends on iron because catalases and peroxidases require heme as cofactor (Schrettl et al., 2007). Therefore, the impact of siderophores on the resistance to oxidative stress was studied. As showed in (Figures 6A,B) deficiency of siderophores (Δ*NrsidA*) caused hypersensitivity to H_2_O_2_ during iron-depleted growth. With the increase of extracellular iron availability, the defect was cured slightly, indicating the major role of siderophore is efficient iron utilization rather than iron detoxification (Figures 6A,B). To further verify the speculation as mentioned above, the effects of hydrogen peroxide on conidial and hyphal survival were tested with these mutants in more details (Figures 6C,D). Whether conidia or hyphae of *N. rileyi* strains killed by hydrogen peroxide, Δ*NrFtrA* mutants were as resistant as WT; nevertheless, Δ*NrSidA* mutants were significantly more sensitive than WT, implying that siderophore played an important role in the oxidative-stress resistance of *N. rileyi* conidia and hyphae.

### *NrSidA* is required for full virulence against *S. litura* host

**Figure 7:**
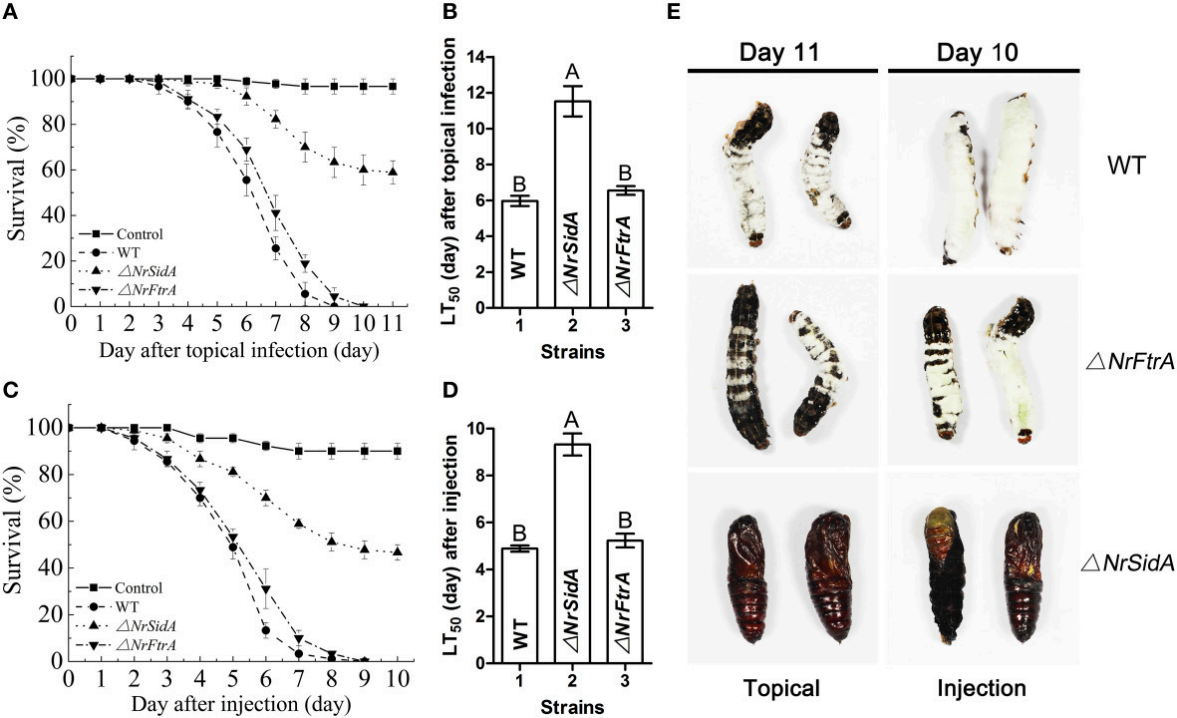
**Insect bioassays. (A)** Survival of *S. litura* larvae following topical infection with the fungal spore suspensions. Control insects were treated with mineral oil. **(B)** LT _50_ for topical infection assay. **(C)** Survival of insect after injection of conidia directly into the insect haemocoel. Sterile water without conidia was used as blank control. **(D)** LT_50_ by injection application. **(E)** Fungal mycosis. *S. litura* larvae were topical infection or injected to death with the conidia of WT and mutants. Shown are mycosis of insect cadavers 11 or 10 day after treatment. Error bars are standard deviations of three trials. Different letters represented significance groups at *P* < 0.01.

To examine the effects of *NrSidA* and *NrFtrA* deletion on the pathogenic ability of *N. rileyi*, insect bioassays by both topical infection and injection of fungal spores into *S. litura* larvae were performed. The values of median lethal time (LT_50_) were estimated and compared among the WT, Δ*NrSiDA* and Δ*NrFtrA* mutants. After topical infection, a significant difference was found between the WT (LT_50_ = 5.97 ± 0.29 days) and Δ*NrSidA* (LT_50_ = 11.52 ± 0.85 days) (*P* < 0.001) but not between the WT and Δ*NrFtrA* (LT_50_ = 6.55 ± 0.24 days) (Figures 7A,C). Similar results were observed in injection assays that the differences between the WT (LT_50_ = 4.89 ± 0.22 days) and Δ*NrSidA* (LT_50_ = 9.32 ± 0.52 days) were significant with no obvious differences between the WT and Δ*NrFtrA* (*P* < 0.001) (Figures 7B,D), demonstrating that it is *NrSidA*, but not *NrFtrA* that is absolutely required for *N.rileyi* virulence.

**Figure 8:**
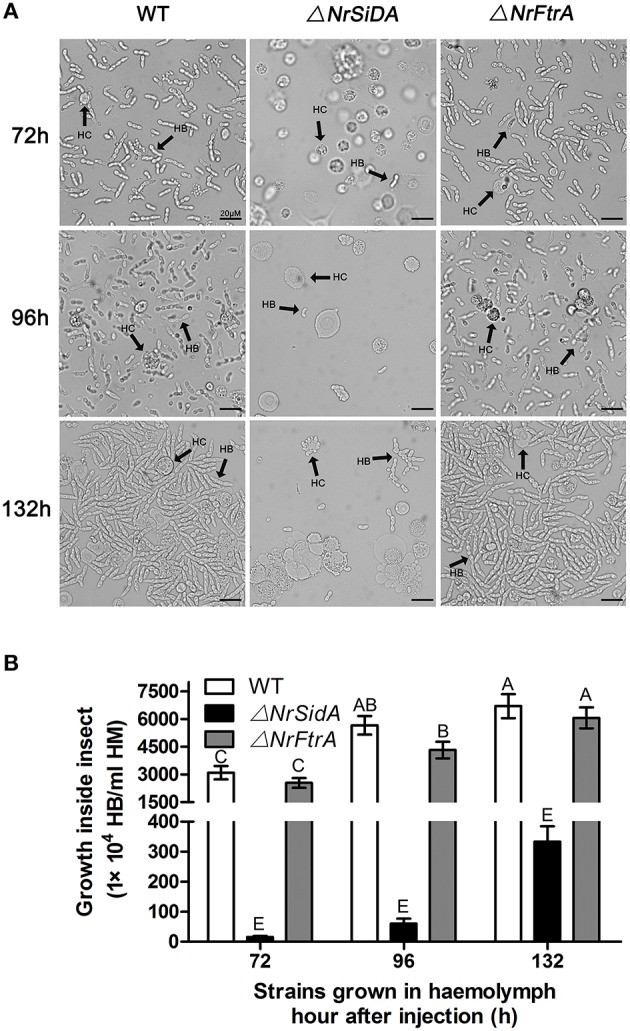
**(A)** Fungal development in insect haemocoels. *S. litura* larvae were injected with the spores of the WT and mutants and bled for microscopic examination of fungal development at 72, 96, and 132 h. **(B)** Concentration of hyphal bodies in insect haemocoels. Fungal cells were counted using a haemocytometer. HC, haemocyte; HB, hyphal body. Bar = 20 μm. Error bars are standard deviations of three trials. Means indicated by the same uppercase letter are not significantly different from one another, *P* < 0.01.

*S. litura* hemolymph was investigated microscopically and hyphal body production was also quantified over time after inoculation with the mutants and WT conidia. Both Δ*NrFtrA* and WT conidia escaped from insect immune and started yeast-type budding to multiple hyphal-bodies 72 h following the injection of fungal spores. However, the Δ*NrSidA* spores were continuously attacked and encapsulated by insect haemocytes and only a few yeast-like cells were observed (Figure 8). After 96 h, Δ*NrSidA* evaded insect immune defense and started to multiply in the insect haemocoel (Figure 8A). After 132 h, Δ*NrSidA* hyphal-bodies in insects increased rapidly but the average number remained 20-fold less than that of WT and Δ*NrFtrA* mutants (Figure 8B). In both bioassays, almost all the *S. litura* larvae died of the infection of the WT and Δ*NrFtrA* strains were before pupation. By contrast, most death by the Δ*NrSidA* strains were in the stage of the pupae (Figure 7E). Furthermore, insect cadavers were fully covered by the WT and Δ*NrFtrA* fungal spores or mycelia post-insect death, but these were not observed in Δ*NrSidA*-treated insects. Thus, abrogation of *N. rileyi* siderophore biosynthesis by *NrSidA* deletion prevents initiation of *S. litura* infection, which cannot be supported by RIA alone. Consistently, inactivation of RIA by deletion of *NrFtrA* is inconsequential for virulence.

### Loss of both *NrSidA* and *NrFtrA* alters iron-related genes expression, but only the *NrSidA* deletion changes the ros detoxification at transcription level during MS development

**Figure 9:**
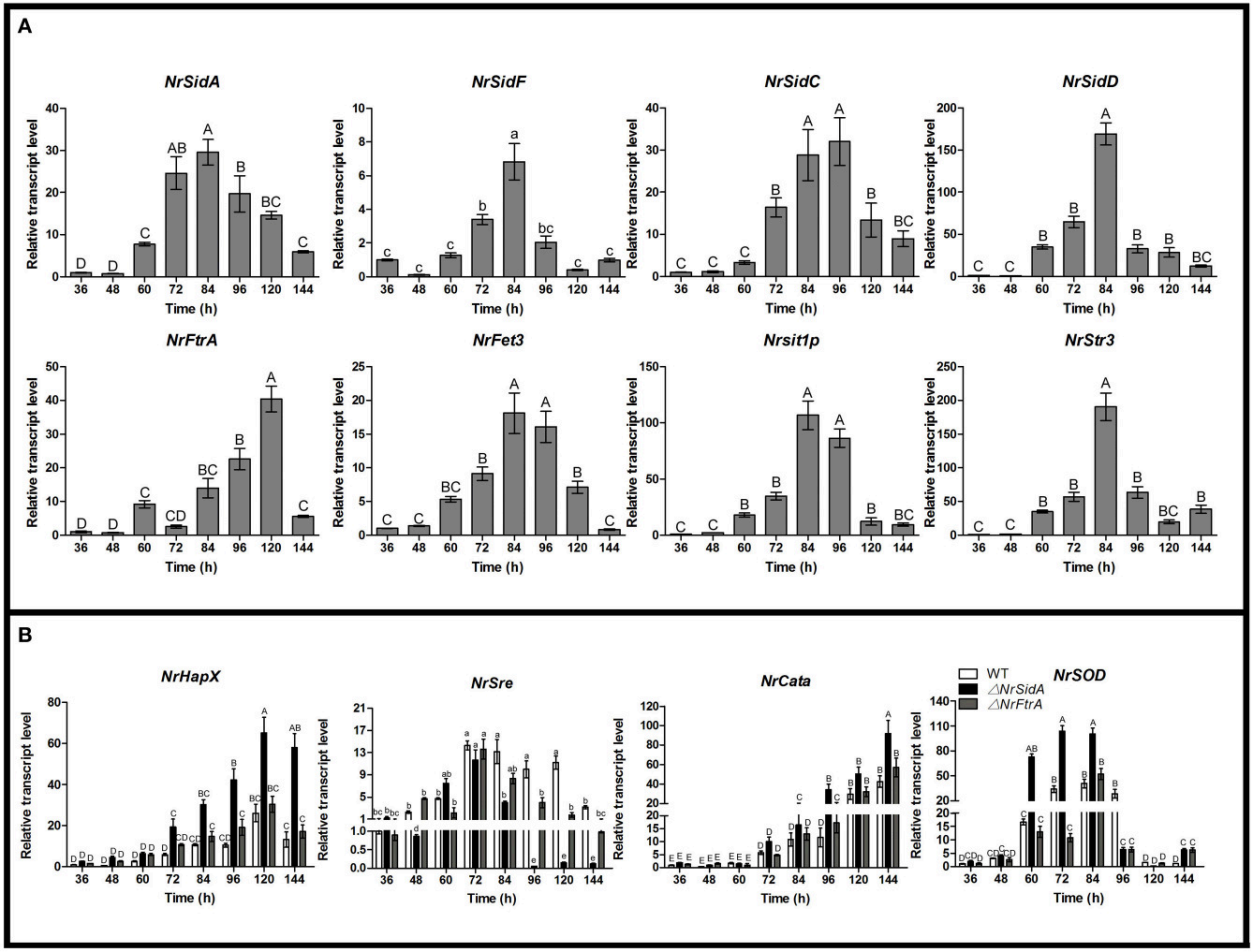
**Relative transcript abundances of iron uptake-related and ROS detoxification related genes during the development of MS. (A)** Relative transcript abundances of iron uptake-related genes were analyzed in WT at different stages of MS formation. **(B)** Relative transcript abundances iron uptake- regulatory genes and ROS-detoxification-related genes were detected in WT and mutants at different stages of MS formation. TEF and TUB transcripts were used as reference. Uppercase or lowercase letters indicated the statistically significant level at *P* < 0.01 or *P* < 0.05, respectively and the same letter were not significantly different from one another. Error bars are standard deviations of three trials.

To better understand the contribution of two high affinity iron uptake pathways required by MS production, we investigated stage-specific expression pattern analysis of related genes during the MS development in WT. The expression trends of the siderophore biosynthesis genes (*NrSidA, NrSidC, NrSidF*, and *NrSidD*) were consistent with that of siderophore transporter genes (*NrSit1p* and *NrStr3*) during the MS formation. During coalescing hyphae time (36 and 48 h), the expression abundances were low, started to increase at MS initiation stage (60 h), peaked at the MS formation stage (72 and 84 h) or MS maturation stage (96 h) and then declined at the late stage (120 and 144 h; Figure 9A). Interestingly, the *NrSidC* expression was highest at MS maturation stage, which was in agreement with the finding that the greatest intracellular iron content in WT was present at 96 h. The analogous expression profiles were found in the RIA genes (*NrFtrA* and *NrFet3*), but the difference was that the higher expression levels occurred at late maturation and at MS maturation stage, implying that RIA mainly took part in iron supplement at the maturation and late maturation. To dive deep into the iron homeostasis by the gene regulation during MS formation, the expression patterns of two iron regulatory genes (*NrHapX* and *NrSre*) were analyzed in the WT and mutants. The transcript abundance of bZIP-type regulator *NrHapX* was enhanced as the MS matured (Figure 9B). It was also worth mentioning that a more significant increase in the Δ*NrSidA* mutants rather than in WT or Δ*NrFtrA* mutants because of a defect of iron uptake caused by the siderophore missing under iron depletion (Figure 3C). As expected, the transcript concentrations of the GATA transcription factor *NrSre* also increased with the augmentation of intracellular iron contents in WT and mutants. In spite of this, a dramatical decreased expression occurred at maturation and late maturation stage of MS in Δ*NrSidA* rather than in Δ*NrFtrA* compared with the WT due to the consumption of iron in AM. As oxidative stress occurred during MS development (Song et al., 2013), the transcript abundances of reactive oxygen species (ROS) detoxification genes (*NrSOD* and *NrCata*) were analyzed in the WT and mutants. The forming of MS increased the transcripts of the *NrCata* and *NrSOD* among the WT and mutants, validating that the production of ROS is along with the formation of MS (Figure 9B). Notably, a dramatically higher transcript level of *NrSOD* appeared in Δ*NrSidA* mutants at both initiation and formation stage of MS compared to the WT and Δ*NrFtrA* mutants, suggesting that a decrease of the intracellular iron by *NrSidA* deletion resulted in a rise of ROS.

**Figure 10:**
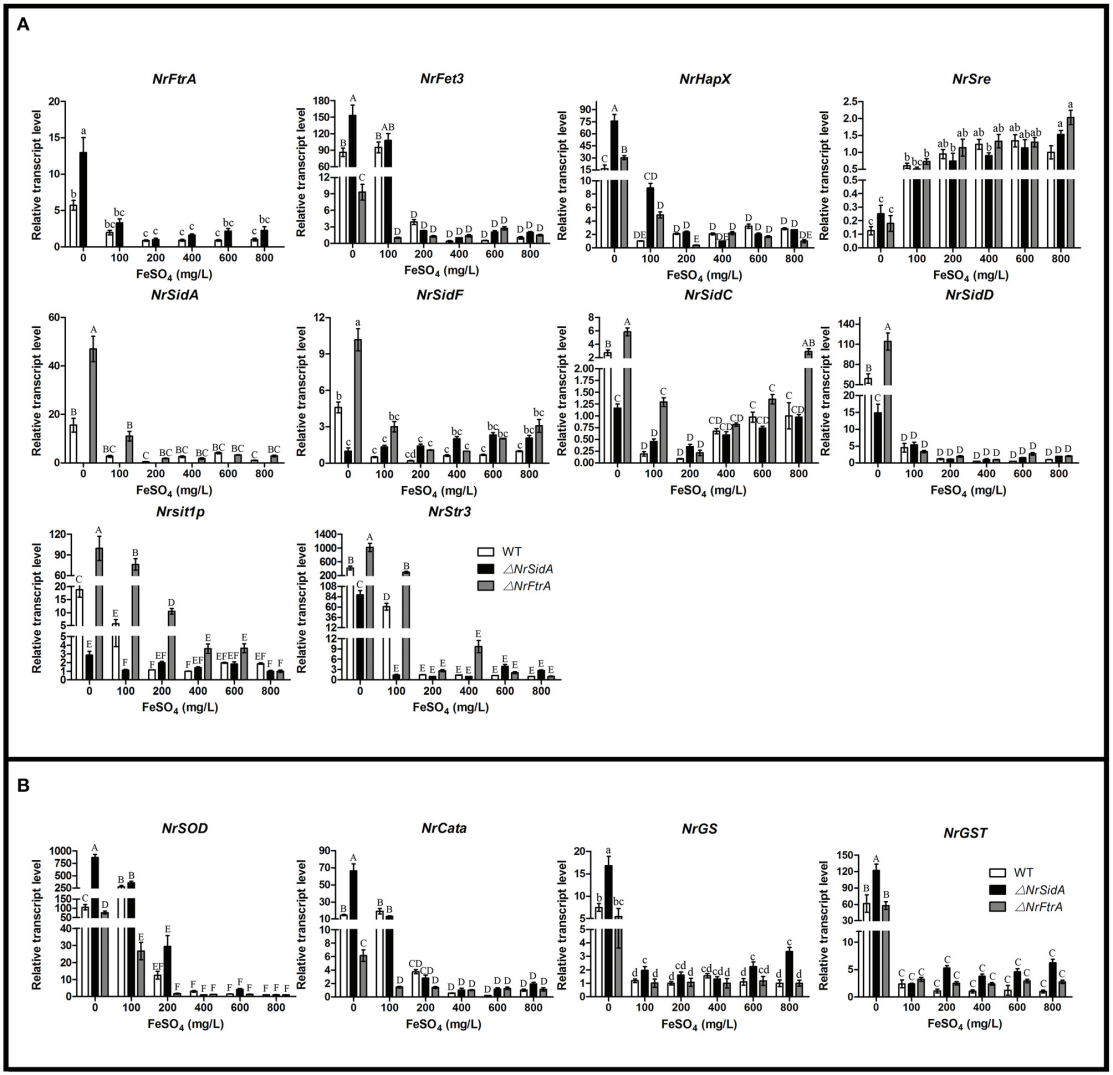
**Relative transcript abundances of iron uptake-related genes (A) and ROS detoxification related genes (B) in 3.5-day-old MS of the WT and mutants at different iron supplies**. TEF and TUB transcripts were used as reference. Uppercase or lowercase letters indicated the statistically significant level at *P* < 0.01 or *P* < 0.05, respectively and the same letter were not significantly different from one another. Error bars are standard deviations of three trials.

The transcriptional levels of the iron uptake related genes and two iron regulatory genes were further investigated under different iron concentrations for MS formation in the WT and mutants. Expression of the siderophore biosynthesis and RIA genes in WT and mutants was strongly repressed under iron supplemented conditions except the intracellular siderophore biosynthesis *NrSidC* which showed a high expression under the high iron concentrations (Figure 10A). Under iron starvation condition, the loss of *NrSidA* led to an obviously increased expression of the RIA related genes in Δ*NrSidA* mutants whereas the *NrFtrA* deletion resulted in evidently higher transcription levels of the siderophore biosynthesis genes in Δ*NrFtrA* mutants (Figure 10A). As expected, transcripts of the regulator *NrHapX* were present at higher concentration in Δ*NrSidA* mutants under iron deletion compared to WT and Δ*NrFtrA* mutants. The repressor *NrSre* was typically negatively regulated by iron. To explore the role of the iron played in the ROS detoxification, the expression levels of the *NrSOD, NrCata*, glutathione synthase (*NrGS*) and glutathione S transferase (*NrGST*) genes were also further detected. Under iron-limiting conditions, a significantly higher transcript concentration was detected in Δ*NrSidA* mutants rather than in Δ*NrFtrA* mutants compared to WT. Increasing concentrations of iron to 400, 600, or 800 mg/L rapidly decreased the transcript concentrations of the *NrSOD, NrCata, NrGS*, and *NrGST* (Figure 10B) in WT and mutants, suggesting that high levels of intracellular iron might promote the detoxification of ROS during MS formation.

To test the hypothesis that iron acquisition deficiency by lack of siderophores led to the observed oxidative stress sensitivity and the higher expression of the ROS detoxification-related genes in Δ*NrSidA* mutants, the expression profiles of the iron uptake related genes were further analyzed when treatment with different concentrations of hydrogen peroxide. The expression of selected genes did not associate with the change of hydrogen peroxide (data not show) with the exceptions of the siderophore transporter genes (*Nrsit1p* and *NrStr3*). They were present at higher concentration only in the WT and Δ*NrFtrA* mutants as the levels of H_2_O_2_ increased (Figure S5).

## Discussion

### Siderophore-mediated iron uptake, but not RIA, is indispensable for pigmented MS development in *N. rileyi*

The necessity of the iron involving in MS biogenesis and the up-regulated expression of some iron acquisition related genes in comparative transcriptome raised the questions of what are the main acquisition strategies to achieve the requirement of iron during MS biogenesis and what roles the iron plays. Firstly, we examined the contribution of two high affinity iron uptake pathways played in this physiological process at transcription level. Stage-specific expression pattern analysis showed that the expression of siderophore biosynthesis related genes, as well as the siderophore transporter genes were strongly upregulated during MS development of entomogenous fungi *N. rileyi* while the higher transcript abundances of RIA related genes were present at the maturation and late maturation stage (Figures 3, 9A). These data along with quantitative analysis of intracellular iron in WT indicated that both iron uptake pathways take part in the MS production but the siderophore-mediated iron acquisition pathway meets the most requirement of iron during the formation and mature period of MS while the RIA just partly compensates the iron at the late maturation.

To understand how two high affinity iron uptake pathways affect the MS formation, *NrSidA*, which catalyzes the first committed step of hydroxamate-type siderophore biosynthesis, and the high affinity iron permease *NrFtrA*, the key permease gene for the reductive iron assimilation, were deleted. The loss of the *NrSidA* led to the defects in pigmented MS development and the similar phenotype also appeared in WT and Δ*NrFtrA* mutants under iron deletion condition (Figure 2A). Combined with the absence of *NrSidA* and changes in intracellular iron and extracellular siderophore of MS demonstrated the lack of the total siderophore caused by *NrSidA* missing impaired cellular iron uptake (Figure 3) and the reduction of the intracellular iron contents in the Δ*NrSidA* mutants contributed to the defect in MS production. These results were further validated with the differences in the expression levels of iron regulatory genes among the WT and both genes mutants. The increased expression levels of *NrHapX* during the MS differentiation and the decreased transcript concentrations of *NrSre* at the maturation and late maturation of MS in the Δ*NrSidA* mutants is a consequence of the lack of adequate intracellular iron (Figure 9B). The increase in the iron concentrations of AM medium fully restored altered biomass; however, it was not enough to overcome reduced pigment (Figures 2D,E). The pigment in the Δ*NrSidA* mutants could be basically recovered by adding of the high concentrations of ferrichrome into AM (results not shown). Likewise, whether in the maize (*Zea mays*) pathogen *Cochliobolus heterostrophus* or the citrus fungal pathogen *Alternaria alternata*, the loss of the extracellular siderophore by a nonribosomal peptide synthetase (NPS6) deletion is defective in pigmentation (Oide et al., 2006; Chen et al., 2013). Loss of SIT1, a siderophore transporter gene, in *Cryptococcus neoformans* exhibits altered melanin deposition and laccase activity (Tangen et al., 2007). It remains uncertain the role of the siderophore plays in the biosynthesis of secondary metabolites. And a reduction in melanin biosynthesis by Δ*NrSidA* mutants could be at least a result of a reduction in iron availability for some melanin biosynthesis enzymes require metal repletion for full activity (Tangen et al., 2007; Choi et al., 2012).

The role of the iron in MS development is suggested by the studies that Fe^2+^ promotes MS biogenesis by catalyzing the formation of hydroxyl radicals that induce sclerotial biogenesis (Georgiou et al., 2006; Papapostolou and Georgiou, 2010; Song et al., 2013, 2014). Nevertheless, detoxification of hydrogen peroxide depends on iron because catalases and peroxidases require heme as cofactor (Haas et al., 2008). The decreased transcript abundances of the *NrCata, NrSOD, NrGS*, and *NrGST* induced by increased iron concentration in AM medium at the 3.5-days old MS indicated that the major role of iron is efficient ROS detoxification rather than the formation of ROS. Furthermore, the higher transcript concentrations of these genes in the Δ*NrSidA* mutants suggested that the lack of adequate intracellular iron by the *NrSidA* missing leads to a defects in ROS effective detoxification. The hypothesis is also supported by the hypersensitivity of the conidia and the hypha in the Δ*NrSidA* mutants to oxidative stress (Figures 6C,D). Some similar findings are also observed in *A. fumigatus, Cochliobolus heterostrophus* and *Magnaporthe grisea* that NrSidA homologs missing makes fungi be hypersensitive to oxidative stress (Schrettl et al., 2007; Hof et al., 2009; Condon et al., 2014). So iron is crucially involved in the destruction of ROS. As iron can potentiate oxidative stress, siderophore might play an instrumental role to avoid iron toxicity, which is proven by that the sharp increase in the transcripts of *NrSidC*, an intracellular siderophore synthetase, were followed by an augment of intracellular iron contents at MS formation stage (Figures 3C, 9A). Iron metabolism is interconnected with the regulatory production of ROS. In *A. fumigatus*, Brandon et al. predicted blocking siderophore-mediated iron uptake reduces resistance to oxidative stress by constructing a Boolean network model (Brandon et al., 2015). Remarkably, Chen et al. also demonstrated in the citrus fungal pathogen *Alternaria alternata* that siderophore-mediated iron acquisition has profound effects on ROS detoxification by regulating the NADPH oxidase, the redox activating yes- associated protein 1 regulator, and the high-osmolarity glycerol 1 mitogen-activated protein kinase (Chen et al., 2014). Here, our findings show that the major role of siderophore is collaborated with iron for the ROS detoxification during the MS biogenesis.

The expression patterns of MS after the treatment of hydrogen peroxide is further evidence that the increase of the ROS requires more iron for detoxification by the upregulated expression of the siderophore transporter genes (Figure 5S). However, a different expression pattern is present in *A. nidulans* (Eisendle et al., 2006) and there is no obvious effects on the expression of siderophore transporter *mirB* after treatment with hydrogen peroxide. The difference may result from that the MS biogenesis is induced by oxidative stress (Georgiou et al., 2006; Song et al., 2013; Liu et al., 2014) and the requirements of iron for ROS detoxification during MS biogenesis is more than the normal for growth (Song et al., 2013).

### Siderophore-mediated iron uptake is required for sporulation, germination and dimorphic switching

In *N. rileyi*, like in the vast majority of fungus analyzed so far, high-affinity iron uptake systems are indispensable not only for hyphal growth under iron-limited conditions, but also for sporulation (Eichhorn et al., 2006; Oide et al., 2006; Schrettl et al., 2007; Figure 4). Under iron-sufficient conditions, vegetative growth rates of these mutant strains did not significantly differ from that of WT but under iron-limiting conditions in the presence of BPS, the Δ*NrSidA* mutants grew significantly slowly (Figure 6A). In contrast, Δ*sidA* of *A. fumigatus* failed to grow under iron deletion conditions with BPS (Schrettl et al., 2007). With respect to conidiation of *N. rileyi*, the Δ*NrSidA* mutants had a marked reduction either on AMM or SMAY medium and was only rescued by addition of a high concentration of iron (1.5 mM FeSO_4_) (Figure 4A). Similarly, both Δ*sid1* mutants of *Colletotrichum graminicola* and Δ*SIDA* mutants of *A. fumigatus* are unable to produce conidia under iron starvation and high iron or corresponding siderophores fully restored conidiation (Schrettl et al., 2004; Wallner et al., 2009; Albarouki et al., 2014). Besides, *SidA* of *A. nidulans* was found to be significantly upregulated during asexual sporulation (Garzia et al., 2013). Thus, growth and sporulation of *N. rileyi* depends on iron steady supplies and siderophore is particularly crucial for iron trafficking. Noteworthily, sporulation in the Δ*NrSidA* mutants could be stimulated only with 1.5 mM FeSO_4_, but not FeCl_3_, indicating the preference on Fe^2+^ in a reductive iron assimilatory system of *N. rileyi*. However, under iron-sufficient or deficient conditions, the Δ*NrFtrA* mutants behaved like WT, suggesting that the loss of reductive iron uptake system was compensated fully by the siderophore-mediated iron acquisition.

In addition, the *NrSidA* deletion in the dimorphic fungus *N. rileyi* prolonged the conversion from yeast-cell to hypha on SMAY medium (Figures 4C,D). The conversion from yeast-cell to hypha contributes to environmental survival and transmission to new hosts (Gauthier, 2015). Several evidences have explained the role of iron acquisition in the yeast-to-hyphal transition. Cell-type-specific transcriptional profiles analysis reveals *sidF* (*NrSidA* orthologue) and *sidD* (*NrSidD* orthologue) are highly expressed in *Penicillium marneffei* yeast cells (Pasricha et al., 2013). Besides, the loss of a vacuolar ATPase, *VMA1*, involved in iron homeostasis in *H. capsulatum*, impairs the mycelial transition at ambient temperature (Hilty et al., 2008). Collectively, iron limitation by the *NrSidA* deletion should have a drastic effect on the morphologic switch.

Conidial siderophore storage was an important germination factor, as germination of conidia fails or is greatly delayed unless a suitable siderophore is supplied due to the loss of cellular siderophores in *A. nidulans, A. fumigatus* and *N. crassa* (Eisendle et al., 2006; Schrettl et al., 2007). Hence, a delay of the germination under iron-sufficient or deficient conditions (Figures 5C,D) and a failure under depletion condition in the presence of BPS in Δ*NrSidA* mutants (Figure 5B) results from the missing of siderophore-mediated iron storage or utilization of intracellular iron (Figure S3B).

### Loss of the *NrSidA*, but not *NrFtrA* is required for full virulence of the *N. rileyi* against *Spodoptera litura*

The research on the roles of siderophores in fungal pathogenicity to host was widespread studied in pathogenic fungi of plant and human with the differences among different pathosystems, while the studies about entomogenous fungal pathogenicity to insect are scarce. In this study, we found that the siderophore biosynthesis, but not reductive iron assimilation is essential for virulence in the *N. rileyi* against *S. litura*. In both topical infection and injection assay, the Δ*NrSidA* mutants showed less virulence in *S. litura* than WT while the Δ*NrFtrA* mutant was as virulent as the wild-type strain (Figure 7). Further investigation revealed that both germination and proliferation of the Δ*NrSidA* mutants conidia in insect haemocoel slowed dramatically (Figure 8). Disruption of siderophore production resulted in iron-dependent growth during the infection and the growth defect in *S. litura* ultimately led to the loss of virulence. By contrast, in *A. fumigatus*, no germination of Δ*sidA* conidia was observed *in vivo*, leading to the total loss of the virulence caused by the *SidA* missing (Schrettl et al., 2004).

In other fungal pathogens, one mode of high-affinity iron acquisition is also favored over the other. In *A. fumigatus*, siderophore biosynthesis is essential for virulence, but RIA is neither necessary nor sufficient for normal growth and survival in the host (Schrettl et al., 2007). Similar results are reported in the phytopathogens *Cochliobolus miyabeanus, Alternaria alternata, Magnaporthe grisea, Fusarium graminearum, Colletotrichum graminicola*, and human pathogen *Histoplasma capsulatum* that extracellular siderophore production is required for full virulence (Greenshields et al., 2007; Hwang et al., 2008; Hof et al., 2009; Chen et al., 2013; Albarouki et al., 2014). However, the loss of the *SID1* gene in *U. maydis* does not affect virulence in maize, instead, RIA is required for virulence (Mei et al., 1993). Analogous observations have been also found in *Candida albicans* and *Cryptococcus neoformans*, where high-affinity iron permease genes are essential for systemic infection (Ramanan and Wang, 2000; Haas et al., 2008). Our findings indicate that *N. rileyi* prefers high affinity siderophore iron acquisition over RIA.

In summary, our studies revealed siderophore-mediated iron uptake is more important than reductive iron uptake. Even though both are induced upon iron deprivation, *NrSidA*, but not *NrFtrA*, is required for pigmented-MS morphogenesis, oxidative-stress resistance, sporulation, phase transition, germination and full virulence of *N. rileyi* against *S. litura*. As a cofactor, iron is necessary for ROS detoxification instead of catalyzing the formation of ROS during the MS formation; siderophore is very important to assist iron mobilization and meanwhile avoid iron toxicity. In view of these findings, a certain amount of iron is indeed an essential part for the MS induction. Thus, a complete understanding of iron uptake strategies during the MS formation in *N. rileyi* at the molecular level provides a valuable insight into the molecular mechanism of MS differentiation and serves as a guidance in industrialized mass production of *N. rileyi*.

## Author contributions

YL conducted the main experiments and wrote the original manuscript. XL provided a lot of help throughout the experiment. RL provided great help to screen the stable transformants. CS provided assistance with fungal transformation. ZS, ZW, and YY provided technical oversight and critical manuscript review and editing. All authors discussed the results and implications and commented on the manuscript at all stages.

### Conflict of interest statement

The authors declare that the research was conducted in the absence of any commercial or financial relationships that could be construed as a potential conflict of interest.

## Abbreviations

AM: liquid-amended medium
AMM: *Aspergillus* minimal medium
ATMT: *Agrobacterium tumefaciens* mediated transformation
BPS: bathophenanthroline disulfonate
CAS: Chrome azurol S
CFW: calcofluor white
CR: congo red
FPNI- PCR: fusion primer and nested integrated PCR
LIP: labile iron pool
LT_50_: the median lethal time
MS: Microsclerotia
PBS: phosphate buffered saline
qRT-PCR: quantitative Reverse-Transcriptase Polymerase Chain Reaction
RIA: reductive iron assimilation
ROS: reactive oxygen species
SMAY: Sabouraud maltose agar with 1% yeast extract
SDS: sodium dodecyl sulfate
SIT: siderophore-iron transporters
TT_50_: the median transition rates time
UMF: Unknown Major Facilitator
WT: wild type.
