# Supplementary figures and images for: Siderophore Biosynthesis but Not Reductive Iron Assimilation Is Essential for the Dimorphic Fungus Nomuraea rileyi Conidiation, Dimorphism Transition, Resistance to Oxidative Stress, Pigmented Microsclerotium Formation, and Virulence
Source: Front Microbiol. 2016 Jun 16;7:931. doi: 10.3389/fmicb.2016.00931 (PMC4909778; doi:10.3389/fmicb.2016.00931)

# D

*NrSidA*

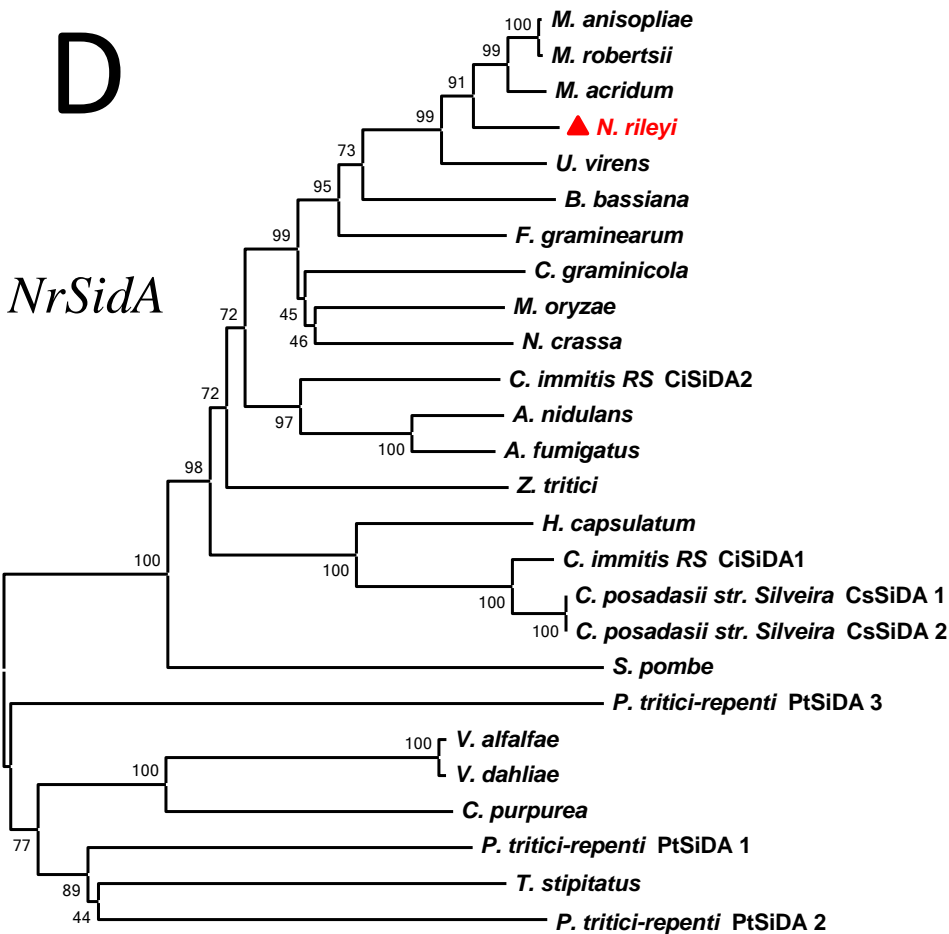

E

*NrFtrA*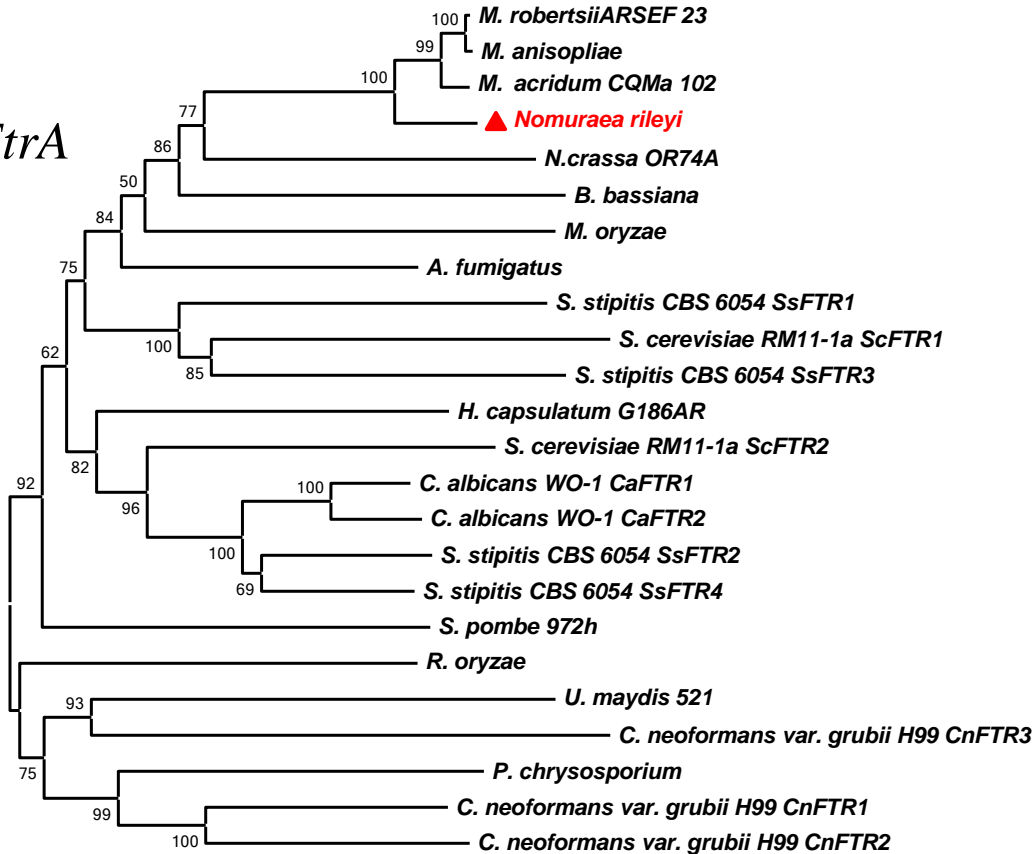

0.05

Supplement: Supplementary file 1 [file Image1.PDF]

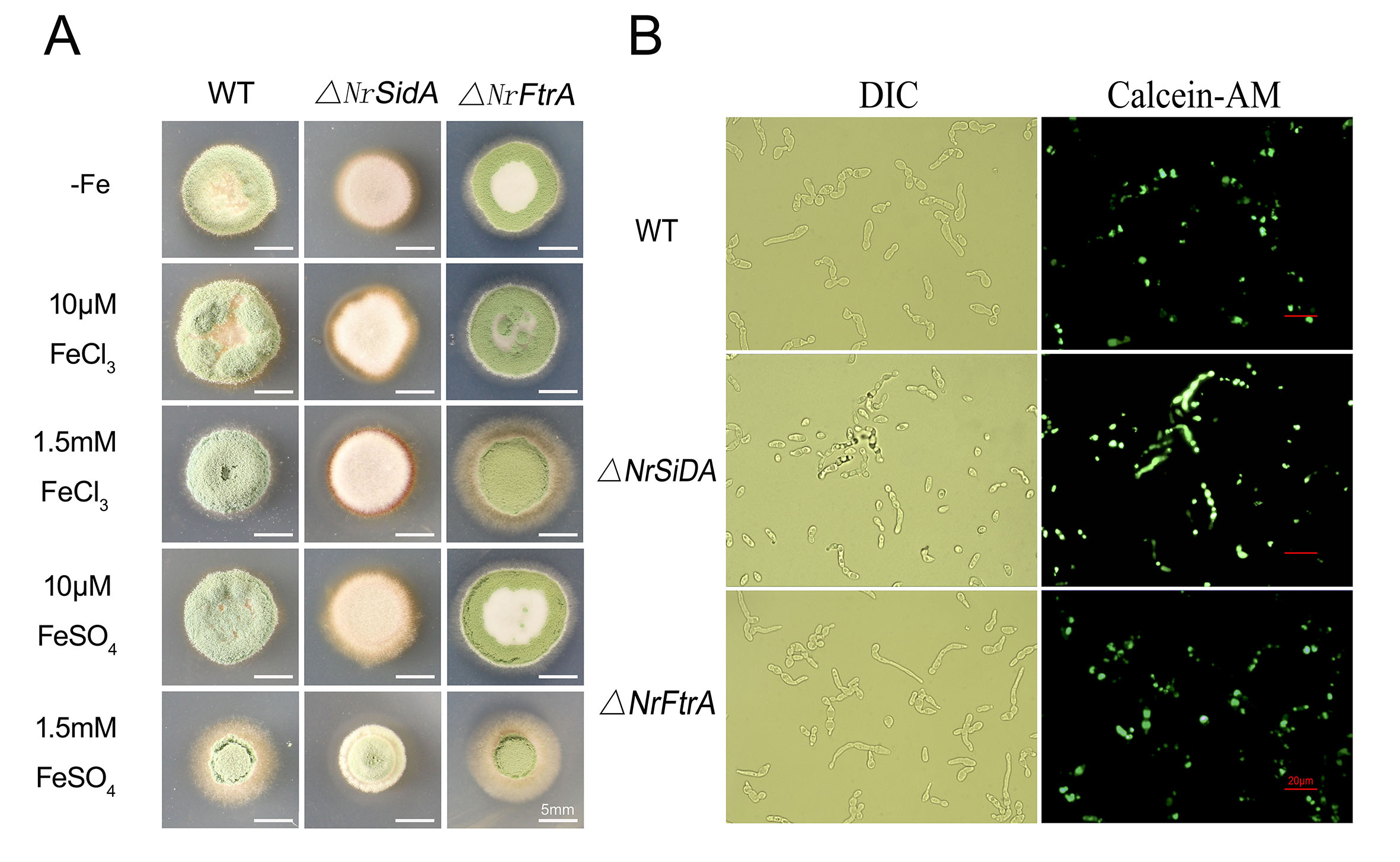

Supplement: Supplementary file 3 [file Image3.JPEG]

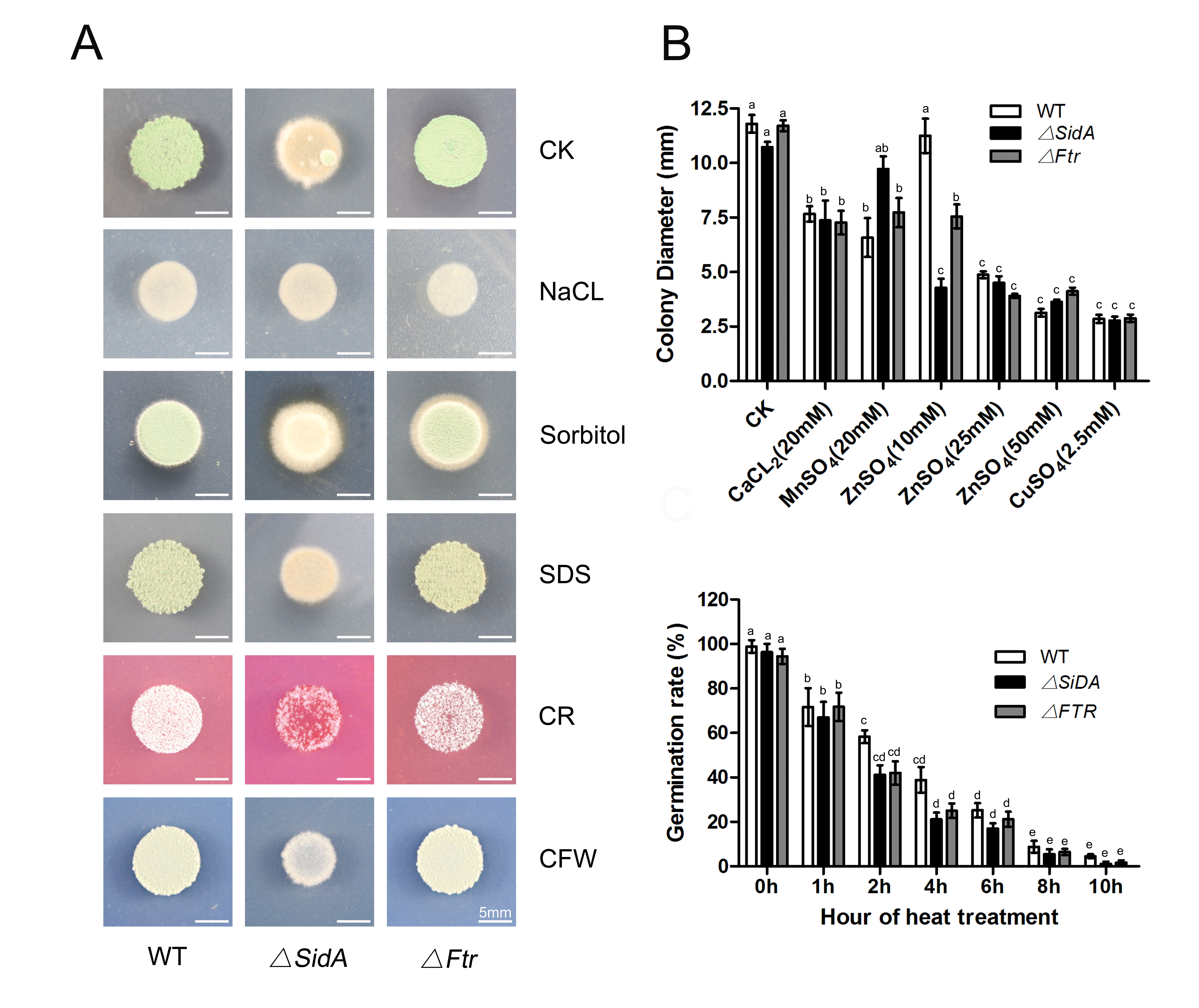

Supplement: Supplementary file 4 [file Image4.JPEG]

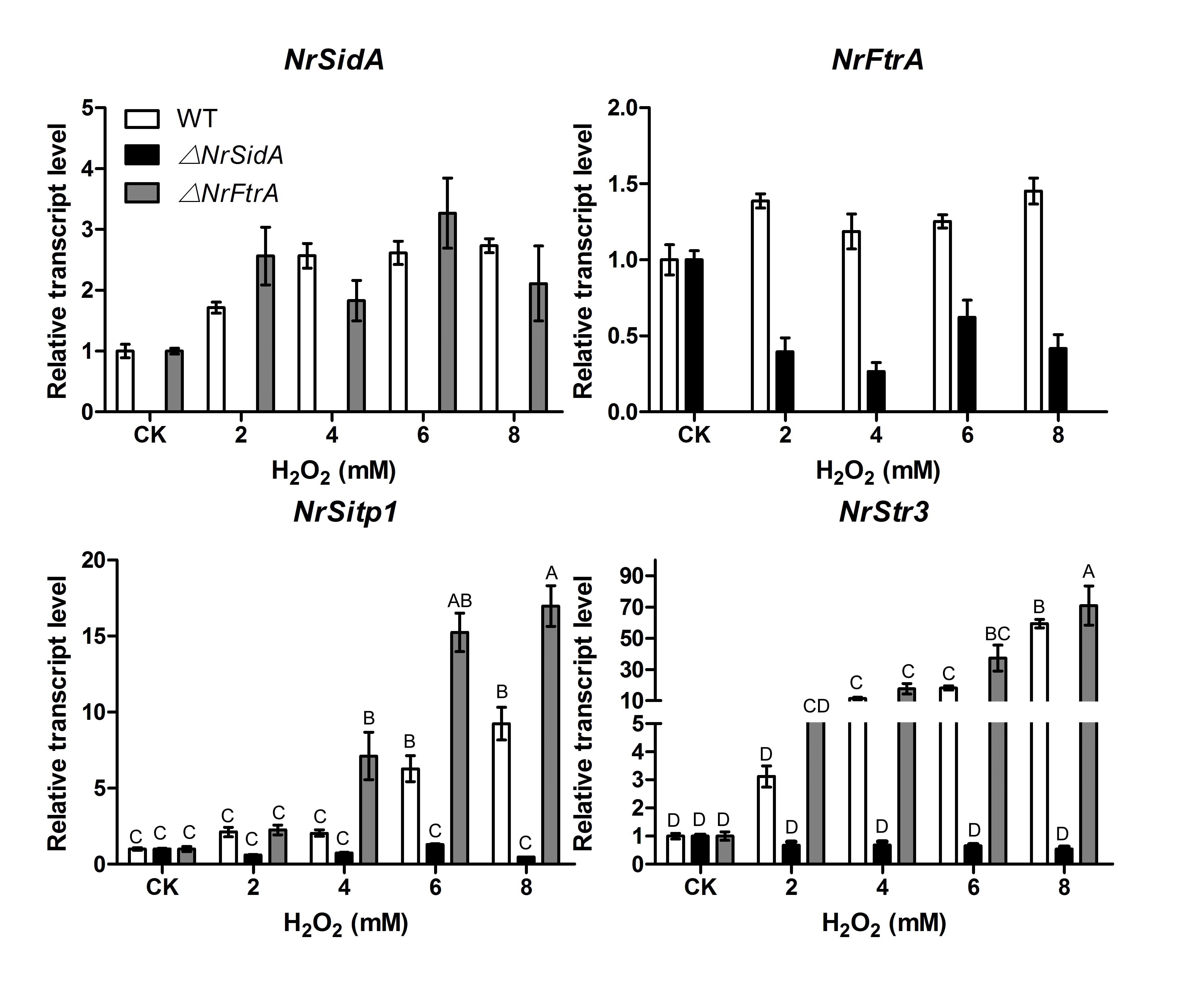

Supplement: Supplementary file 5 [file Image5.JPEG]

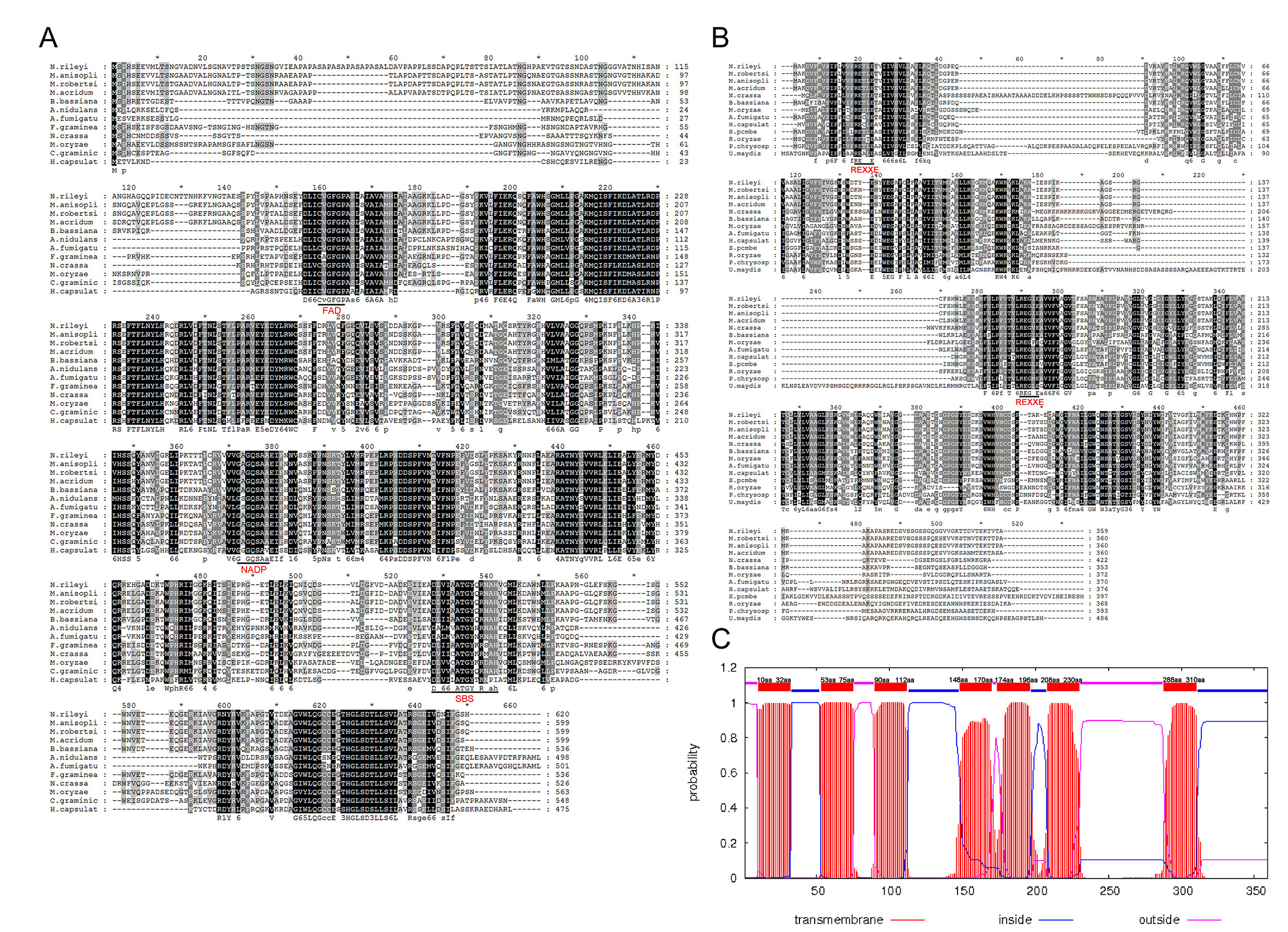

Supplement: Supplementary file 6 [file Image6.JPEG]
