# Supplementary material for: Siderophore Biosynthesis but Not Reductive Iron Assimilation Is Essential for the Dimorphic Fungus Nomuraea rileyi Conidiation, Dimorphism Transition, Resistance to Oxidative Stress, Pigmented Microsclerotium Formation, and Virulence
Source: Front Microbiol. 2016 Jun 16;7:931. doi: 10.3389/fmicb.2016.00931 (PMC4909778; doi:10.3389/fmicb.2016.00931)
Supplement: Supplementary file 7 [file Table1.DOCX]

Table S1 Primers used in this study.

| **Name** | **Sequence 5’--3’** | | **Remarks** |
| --- | --- | --- | --- |
| NrSidALBR1 | GGTAAGAAAGGAACAGTCCCGCGG | NrSidA KO left ﬂank chromosome walking | |
| NrSidALBR2 | CCTGTTCTATGCCAAGCACACTCC | NrSidA KO left ﬂank chromosome walking | |
| NrSidALBR3 | GCAATCAACACAGGGCAGGTTGGCC | NrSidA KO left ﬂank chromosome walking | |
| NrSidALBR4 | GCTGGTCCCATTGCATGTATCTTC | NrSidA KO left ﬂank chromosome walking | |
| NrSidALBR5 | AGCGGTTGATTTGATTGTGCGACCC | NrSidA KO left ﬂank chromosome walking | |
| NrSidALBR6 | AAGTAGTCATCTCGCTCGCGTCATC | NrSidA KO left ﬂank chromosome walking | |
| NrSidARBF1 | GAGTTCAGCAAGGGCATCTCGG | NrSidA KO right ﬂank chromosome walking | |
| NrSidARBF2 | AATTTGCTCCAGGAACCGTTACCG | NrSidA KO right ﬂank chromosome walking | |
| NrSidARBF3 | ATTGTGGACAACATCTTTGGCAGCC | NrSidA KO right ﬂank chromosome walking | |
| NrFtrLBR1 | ACCATGCAGAGGAAGAAACC | NrFtr KO left ﬂank chromosome walking | |
| NrFtrLBR2 | GTACGCCTTCACATCTTGCTCCG | NrFtr KO left ﬂank chromosome walking | |
| NrFtrLBR3 | CGTTTCCAGTGTCTCTCGAAAGACG | NrFtr KO left ﬂank chromosome walking | |
| NrFtrLBR4 | GCAAAGGTCTGTTGGCTGAAAG | NrFtr KO left ﬂank chromosome walking | |
| NrFtrLBR5 | TCACAAGGGAAAAGATACGGAGG | NrFtr KO left ﬂank chromosome walking | |
| NrFtrLBR6 | CCAACTGCAAACAGACATCCAGGCC | NrFtr KO left ﬂank chromosome walking | |
| NrFtrRBF1 | ATCTTCGTCACGGTCGGCTT | NrFtr KO right ﬂank chromosome walking | |
| NrFtrRBF2 | GGGAAGACAGCGTCAGCAGCGG | NrFtr KO right ﬂank chromosome walking | |
| NrFtrRBF3 | AGACGACTGATGTGACGGAGAAGAC | NrFtr KO right ﬂank chromosome walking | |
| NrFtrRBF4 | ATCGCTGAACATAATCCAACA | NrFtr KO right ﬂank chromosome walking | |
| NrFtrRBF5 | TCAAGTTCAAGCCTCTGGTGTCC | NrFtr KO right ﬂank chromosome walking | |
| NrFtrRBF6 | GCAGTGAATAGCTCCCTCGCCATTTT | NrFtr KO right ﬂank chromosome walking | |
| NrFtrRBF7 | AGCCCACAGCTTGTATGGTGCTTGT | NrFtr KO right ﬂank chromosome walking | |
| NrFtrRBF8 | GTGCTGGAGTCGTAGCCGAGTTTGC | NrFtr KO right ﬂank chromosome walking | |
| NrSidALF | AGTGAATTCGGGTCGCACAATCAAATCAA | NrSidA KO left ﬂank | |
| NrSidALR | TAA*CTCGAG*GTCTGCTTTGGCTCCTTCAA | NrSidA KO left ﬂank | |
| NrSidARF | ATCTCTAGACGGCTAACAGCGTCTAGTTG | NrSidA KO right ﬂank | |
| NrSidARR | CGTAAGCTTAAAGACAAACAAGCGGGAAG | NrSidA KO right ﬂank | |
| NrFtrLF | ATG*GGATCC*ACACCCTTTCGAGAAGCAAG | NrFtr KO left ﬂank | |
| NrFtrLR | CGC*TCTAGA*AACCAGCAAGACTCTGCTCA | NrFtr KO left ﬂank | |
| NrFtrRF | TAC*CTGCAG*TCAAGTTCAAGCCTCTGGTG | NrFtr KO right ﬂank | |
| NrFtrRR | GTC*CTGCAG*CTTTGCCAATCTTCTCACGA | NrFtr KO right ﬂank | |
| NrSidAScreenF | GACGCGAGCGAGATGACTACT | KO screening *△NrSidA*. Anneals outside the targeted region | |
| NrSidAScreenR | TTGTCCGTCAGGACATTGTTGG | KO screening*△NrSidA*.Anneals within fragment replaced by the hph gene | |
| NrSidA-ORF-F | CCGACAATGTGCTGAGTGG | KO screening *△NrSidA*. Anneals inside the targeted region | |
| NrSidA-ORF-R | CGCCGACAACTACTACACGA | KO screening *△NrSidA*. Anneals inside the targeted region | |
| NrFtrAScreenF | ACGGCCAGTCTGCGCTCTATC | KO screening*△NrFtr*.Anneals outside the targeted region | |
| NrFtrAScreenR | TTGTCCGTCAGGACATTGTTGG | KO screening *△NrFtr*. Anneals within fragment replaced by the hph gene | |
| NrFtrA-ORF-F | GCTTCCCGACACACTCACTA | KO screening *△NrFtrA*. Anneals inside the targeted region | |
| NrFtrA-ORF-R | GGATTATGTTCAGCGATATAGCG | KO screening *△NrFtrA*. Anneals inside the targeted region | |
| NrSidAtzF | CGGCTAACAGCGTCTAGTTG | DIG-labeled probe for △NrsidA | |
| NrSiAtzR | CTCTACGTGCCACAGATTCC | DIG-labeled probe for △NrsidA | |
| NrFtrtzF | AGGTGGGACCTCCGTATCTT | DIG-labeled probe for △NrFtr | |
| NrFtrtzR | AACCAGCAAGACTCTGCTCA | DIG-labeled probe for △NrFtr | |
| NrSidADF | TCAGAGATCCACGGTCAGAG | NrSidA, RT-qPCR | |
| NrSidADR | CGAAGAATGAAGAGCACCAA | NrSidA, RT-qPCR | |
| NrFtrDF | TGCCTTCTGTATCTCGTTGC | NrFtr, RT-qPCR | |
| NrFtrDR | TGCCAGACACTCTTGTCGAT | NrFtr, RT-qPCR | |
| NrSidCDF | TCCAGTCCTTCAGCCTTCTT | NrSidC, RT-qPCR | |
| NrSidCDR | TGCCATCAGGTTTCATGACT | NrSidC, RT-qPCR | |
| NrSidDDF | GCTTCCTCAAAGCAAGGAAC | NrSidD, RT-qPCR | |
| NrSidDDR | TGCAAGGACAATTCCATCAT | NrSidD, RT-qPCR | |
| NrSidFDF | AGCTACACGTCCGACTTCCT | NrSidF, RT-qPCR | |
| NrSidFDR | TGACCCAGTACAGCTCGAAG | NrSidF, RT-qPCR | |
| NrSit1pDF | CCCTTTGTGATCAACACCTG | NrSit1p, RT-qPCR | |
| NrSit1pDR | AGCGAGCAGATGGGATAGAT | NrSit1p, RT-qPCR | |
| NrStr3DF | TAAATATCTTCCCGCCAAGG | NrStr3, RT-qPCR | |
| NrStr3DR | TAGCACCCTTCTTCATGTCG | NrStr3, RT-qPCR | |
| NrSODDF | AATGCCCAGATCCAAGCTAC | NrSOD, RT-qPCR | |
| NrSODDR | TGATGGTGTTGTGAGGAGGT | NrSOD, RT-qPCR | |
| NrCataDF | ATCGACGAGGATCAAGCTCT | NrCata, RT-qPCR | |
| NrCataDF | GCCAACTCTTCGGGTATGAT | NrCata, RT-qPCR | |
| NrBiCataDF | ACTGGAAGCTCAACAACCCT | NrBiCata, RT-qPCR | |
| NrBiCataDR | GCGCATCTGGACATTCTTTA | NrBiCata, RT-qPCR | |
| NrGSF | AAGAAGCAGCTGGACAAGGT | glutathione synthase, RT-qPCR | |
| NrGSR | AAACACCGAAAGAGGAGGAG | glutathione synthase, RT-qPCR | |
| NrGSTF | GGAGGTACTTGGACCGGATA | glutathione S transferase RT-qPCR | |
| NrGSTR | TACGGTTTCCTTTCCGTCTC | glutathione S transferase RT-qPCR | |
