# Supplementary material for: Siderophore Biosynthesis but Not Reductive Iron Assimilation Is Essential for the Dimorphic Fungus Nomuraea rileyi Conidiation, Dimorphism Transition, Resistance to Oxidative Stress, Pigmented Microsclerotium Formation, and Virulence
Source: Front Microbiol. 2016 Jun 16;7:931. doi: 10.3389/fmicb.2016.00931 (PMC4909778; doi:10.3389/fmicb.2016.00931)
Supplement: Supplementary file 8 [file Presentation1.PDF]

Figure S1: Sequence analysis of the *NrSidA* and the *NrFtrA* genes. (A) Amino acid sequence alignment of the putative L-ornithine-N^5-monooxygenase of *N. rileyi* with homologues from other species: *M. acridum*, *M. oryzae, B. bassiana, A. nidulans*, *A. fumigatus, H. capsulatum, F. graminearum, C. graminicola, N. crassa, M. anisopliae* and *M. robertsii.* FAD: A flavin adenine dinucleotide binding domain; NADP: a putative nicotinamide adenine dinucleotide phosphate binding domain; SBS: a conserved putative binding sites for the substrate. (B) Amino acid sequence alignment of the putative the high affinity iron permease gene of *N. rileyi* with homologues from other species: *B.bassiana*, *M. robertsii ARSEF* 23, *M. anisopliae*, *U. maydis* 521, *R. oryzae*, *A. fumigatus*, *P. chrysosporium*, *N. crassa* OR74A, *M. oryzae*, *S. pombe* 972h, *H. capsulatum* G186AR, *C. albicans* WO-1, *S. cerevisiae* RM11, *C. neoformans* var. grubii H99 and *S. stipitis* CBS 6054. REXXE: a conserved putative binding site for iron. Conserved amino acids are indicated in black (identical) and gray (similar). Protein sequences were aligned using ClustalX. (C) Transmembrane structure analysis of NrFtrA proteins using TMHMM-2.0 software. (D) The phylogenetic tree indicates close relatedness of the ornithine N^5-monooxygenase NrSidA of *N. rileyi* with other ornithine N^5-monooxygenases of fungi. With the exception of *Pyrenophora tritici-repentis* (containing three monooxygenases), *Coccidioides immitis* and *Coccidioides posadasii* (containing two monooxygenases) all other fungi shown here contain one single monooxygenase. (E) The phylogenetic tree indicates close relatedness of NrFtrA of *N. rileyi* with other high affinity iron permease proteins of fungi. With the exception of *Scheffersomyces stipitis* (containing four high affinity iron permease) and *Cryptococcus neoformans var. grubii* (containing three high affinity iron permease), *Candida albicans* and *Saccharomyces cerevisiae* (containing two high affinity iron permease), all other fungi shown here contain one single high affinity iron permease. Phylogenetic analysis of the L-ornithine-N5- monooxygenase and high affinity iron permease with MEGA7.0. Numbers above or below branches in D and E indicate percent of bootstrap support when bootstraps are >50% for each clade, performed with 1000 repetitions.

Accession numbers of SidA proteins. *Metarhizium acridum CQMa 102* (EFY85959.1), *Metarhizium anisopliae* (KFG82814.1), *Metarhizium robertsii* ARSEF 23 (XP_007818080.1), *Beauveria bassiana* ARSEF 2860 (XP_008598340.1), *Magnaporthe oryzae* 70-15 (XP_003719605.1), *Aspergillus nidulans* (AAP56238.1), *Aspergillus fumigatus*(AAT84594.1), *Histoplasma capsulatum* (ACC64454.1), *Fusarium graminearum* PH-1 (XP_385547.1), *Colletotrichum graminicola* M1.001 (EFQ31396.1), *Coccidioides posadasii str. Silveira* (EFW21588.1, EFW21588.1), *Coccidioides immitis RS* (XP_001247661.1, XP_001247172.1) *Zymoseptoria tritici* IPO323 (XP_003855779.1), *Schizosaccharomyces pombe* (CAB72228.1), *Neurospora crassa* OR74A (XP_960300.2), *Ustilaginoidea virens* (KDB17608.1), *Claviceps purpurea* 20.1(CCE29720.1), *Talaromyces stipitatus* ATCC 10500 (XP_002478751.1), *Verticillium alfalfae* VaMs.102 (EEY23902.1), *Verticillium dahliae* VdLs.17 (EGY14149.1), *Pyrenophora tritici-repentis* (XP001938606.1; XP001942089.1; XP001932013.1).

Accession numbers of FtrA proteins. *Beauveria bassiana* D1-5(KGQ08864.1), *Metarhizium robertsii* ARSEF 23(XP_007825037.1), *Metarhizium acridum* CQMa 102(XP_007813585.1), *Metarhizium anisopliae* (KFG80659.1), *Ustilago maydis* 521 (DAA04933.1), *Rhizopus oryzae* (AAQ24109.1), *Aspergillus fumigatus* (AAT84596.1), *Phanerochaete chrysosporium* (ABE60665.1), *Neurospora crassa* OR74A (EAA26598.1), *Magnaporthe oryzae* 70-15 (EHA56318.1), *Schizosaccharomyces pombe* 972h (Q09919.1), *Histoplasma capsulatum* G186AR (EEH02984.1), *Candida albicans* WO-1 (EEQ41854.1; EEQ41845.1), *Saccharomyces cerevisiae* RM11-1a(EDV11905.1; EDV08971.1), *Cryptococcus neoformans var. grubii* H99 (XP_012053227.1; XP_012047832.1; XP_012047182.1), *Scheffersomyces stipitis* CBS 6054 (ABN68703.2; ABN67490.2; ABN65004.2; EAZ63978.1).

Figure S2: Targeted disruption of *N. rileyi* NrSidA and NrFtrA. The disruption strategy for NrSidA (A) or NrFtrA (B) showing the native (top) and recombinant (bottom) genomic regions of the respective genes. Restriction sites for genomic DNA/hygromycin resistance cassette ligation and for Southern digestion are shown.(C,D) Southern blots of *△NrSidA* or *△NrFtrA*. *N. rileyi* (wild-type) or putative *△NrSidA* and *△NrFtrA* mutants were digested with XhoI and PstI or BamHI and HindIII, respectively, separated on a 0.8% agarose gel, transferred to a nylon membrane. The expected fragment sizes are indicated on the left.

Figure S3 (A) Morphology of the *N. rileyi* WT and mutants grown on the AMM-Fe medium at the different iron supplies for 14days at 25℃. (B) Free intracellular iron was measured with fluorescence microscope using the fluorescent iron-binding dye calcein-AM in WT, *△NrSidA* and *△NrFtrA N. rileyi*. Red bars represent 20 μm.

Figure S4 Stress tolerance of the WT and mutants to non-biotic stress. (A) Morphology of the *N. rileyi* WT and mutants grown on the AMM medium with 30μM iron under non-biotic stress conditions, including 1M NacL, 1M sorbitol, 10% SDS, 300μM Congo Red (CR) and 20μg/L calcofluor white (CFW).(B) Impact of *NrSidA* and *NrFtrA* deletion on Resistance to the high salinity. 5×10^5 conidia of the WT and mutants were point inoculated and colony diameter was measured after 14 days at 25℃ on AMM medium treated with various high salts. (C) The resistance of the WT and mutants conidia to heat. Lowercase: significant difference, $p < 0.05$, uppercase: significant difference, $p < 0.01$.

Figure S5 Transcript abundance analysis of iron-uptake-related genes in the 3.5-days old MS treated with different concentrations of H_2O_2 for 30min. TEF and TUB

transcripts were used as reference. Uppercase or lowercase letters indicated the statistically significant level at $P<0.01$ or $P<0.05$, respectively and the same letter were not significantly different from one another. Error bars are standard deviations of three trials.
